# The Kelvin and Temperature Measurements

**DOI:** 10.6028/jres.106.006

**Published:** 2001-02-01

**Authors:** B. W. Mangum, G. T. Furukawa, K. G. Kreider, C. W. Meyer, D. C. Ripple, G. F. Strouse, W. L. Tew, M. R. Moldover, B. Carol Johnson, H. W. Yoon, C. E. Gibson, R. D. Saunders

**Affiliations:** National Institute of Standards and Technology, Gaithersburg, MD 20899-0001

**Keywords:** acoustic thermometry, blackbody sources, calibrations, gas thermometry, Johnson noise thermometry, Kelvin, pyrometers, radiation thermometry, SPRTs, thermocouples

## Abstract

The International Temperature Scale of 1990 (ITS-90) is defined from 0.65 K upwards to the highest temperature measurable by spectral radiation thermometry, the radiation thermometry being based on the Planck radiation law. When it was developed, the ITS-90 represented thermodynamic temperatures as closely as possible. Part I of this paper describes the realization of contact thermometry up to 1234.93 K, the temperature range in which the ITS-90 is defined in terms of calibration of thermometers at 15 fixed points and vapor pressure/temperature relations which are phase equilibrium states of pure substances. The realization is accomplished by using fixed-point devices, containing samples of the highest available purity, and suitable temperature-controlled environments. All components are constructed to achieve the defining equilibrium states of the samples for the calibration of thermometers. The high quality of the temperature realization and measurements is well documented. Various research efforts are described, including research to improve the uncertainty in thermodynamic temperatures by measuring the velocity of sound in gas up to 800 K, research in applying noise thermometry techniques, and research on thermocouples. Thermometer calibration services and high-purity samples and devices suitable for “on-site” thermometer calibration that are available to the thermometry community are described. Part II of the paper describes the realization of temperature above 1234.93 K for which the ITS-90 is defined in terms of the calibration of spectroradiometers using reference blackbody sources that are at the temperature of the equilibrium liquid-solid phase transition of pure silver, gold, or copper. The realization of temperature from absolute spectral or total radiometry over the temperature range from about 60 K to 3000 K is also described. The dissemination of the temperature scale using radiation thermometry from NIST to the customer is achieved by calibration of blackbody sources, tungsten-strip lamps, and pyrometers. As an example of the research efforts in absolute radiometry, which impacts the NIST spectral irradiance and radiance scales, results with filter radiometers and a high-temperature blackbody are summarized.

## Introduction

This paper gives a brief review of the realization of the kelvin at the National Institute of Standards and Technology (NIST) and of current research and other activities in thermometry. (From 1934 to 1988, NIST was known as the National Bureau of Standards (NBS), and from 1903 to 1934 it was known as the Bureau of Standards (BS); from 1901 to 1903, it was known as the National Bureau of Standards.) The paper is in two parts. Part I concerns contact thermometry and the realization of the International Temperature Scale of 1990 (ITS-90) [1] at temperatures below 1235 K. Part II concerns non-contact (radiation) thermometry and the realization of the ITS-90 at temperatures above 1234 K.

NIST has been involved in the field of thermometry since shortly after the creation of NBS, and laboratory notebooks detailing calibrations of liquid-in-glass thermometers date back to 1904. Similarly, notebooks concerning calibrations of thermocouples date to 1909 and work on platinum resistance thermometers dates back to 1907. Thus, temperature, one of the SI quantities for which NIST has the responsibility for disseminating its measurement unit—the kelvin—to U.S. industry, has been a feature of the NIST work throughout most of the existence of the organization.

## Part I. Contact Thermometry

### 1. Introduction

The quantity that is designated thermodynamic temperature is defined by the laws of thermodynamics; it is indicated by the symbol *T*, and has the unit kelvin, symbol K. The unit of thermodynamic temperature is defined to be the fraction 1/273.16 of the thermodynamic temperature of the triple point of water. It is common practice to express temperatures in terms of their differences from 273.15 K, the value for the ice point. A thermodynamic temperature *T* expressed in this manner is known as a Celsius temperature *t*, which is defined by the equation

t/°C=T/K−273.15. (1)

The unit of Celsius temperature is the degree Celsius, symbol °C. The magnitude of the degree Celsius is defined to be the same as that of the kelvin. Measures of temperature that are defined to be consistent with the laws of thermodynamics are said to be thermodynamic temperatures. Thermodynamic temperatures, however, are very difficult to measure precisely and accurately. Consequently, internationally-agreed scales of temperature, with temperatures on the scale as close to thermodynamic temperatures as possible at the time the scales are approved, are used to approximate the thermodynamic temperature. These international temperature scales are defined in terms of fixed points, vapor pressures of some liquefied gases, thermometers that can be measured very precisely and fairly easily, and equations that relate measurements of these thermometers to temperatures of the scale.

The Thermometry Group of NIST has the responsibility to develop, establish, and maintain the standards for temperature measurements in the region of contact thermometry (below 1235 K) that are necessary for the Nation’s industrial and scientific progress and, in cooperation with other national laboratories, help establish international uniformity in temperature measurements and promulgate the adopted International Temperature Scale (ITS). To meet this responsibility, members of the staff conduct research on the improvement of thermometry and provide thermometry information to various national and international thermometry standards committees, e.g., the Consultative Committee on Thermometry (CCT) of the International Committee of Weights and Measures (CIPM), the International Electrotechnical Commission (IEC), the American Society for Testing and Materials (ASTM), and the American Society of Mechanical Engineers (ASME). The national thermometry community is informed of the international standards and methodology of measurements by publications, consultations, calibration services, workshops, and thermometry seminars.

This portion of this centennial article gives an overview of some of the efforts in contact thermometry at NIST. Developments at NBS/NIST that are important to thermometry, but not covered, include, e.g., the purification of platinum, the Mueller Bridge (widely used before the modern bridges were developed), purification by slow crystallization and zone refining, cryoscopic determination of purity of substances, ac bridge measurement of resistance, electronics and computers, and many other areas. For those who are interested, lists of NIST publications are available from the NIST Office of Information Services, and those who are interested in publications on thermometry may contact the authors.

### 2. Thermodynamic Temperature

Ultimately all physical properties should be referable to thermodynamic temperature. Thermodynamic temperatures can be accurately determined by:

1. Pressure volume (PV) gas thermometry
2. Velocity of sound (acoustic) gas thermometry
3. Noise thermometry
4. Total radiation thermometry

and related methods, such as Boltzmann distribution of population of energy levels and spectroscopic techniques. Research projects involving all four methods have been conducted at NIST. Thermodynamic temperature measurements are difficult and time consuming and require dedicated effort. Some of these are discussed in sections below.

### 3. International Temperature Scales

A conveniently and accurately reproducible international temperature scale is indispensable for international commerce and exchange of scientific and technical information. Since the late nineteenth century, there has been a series of internationally recognized temperature scales. Those scales are

Chappuis’ constant volume hydrogen gas thermometer scale made available in 1887 through mercury thermometers and referred to as *échelle normale* (NHS) [2];

International Temperature Scale of 1927 (ITS-27) [3];

International Temperature Scale of 1948 (ITS-48) [4];

1958 ^4^He Vapor Pressure Scale of Temperature [5];

International Practical Temperature Scale of 1948. Text Revision of 1960 [IPTS-48(60)] [6];

1962 ^3^He Vapor Pressure Scale of Temperature [7];

The International Practical Temperature Scale of 1968 (IPTS-68) [8];

The International Practical Temperature Scale of 1968. Amended Edition of 1975 [IPTS-68(75)] [9];

The 1976 Provisional 0.5 K to 30 K Temperature Scale (EPT-76) [10];

The International Temperature Scale of 1990 (ITS-90) [1]; and

The 2000 Provisional 1 mK to 1.7 K Temperature Scale [11].

Table 1 lists the fixed points and their assigned temperatures of all the International Temperature Scales that have been adopted. Except for the superconductive transition points, the fixed points are phase equilibrium states of pure substances.

The NHS was based on *verre dur* (hard glass) mercury thermometers that had been compared to the *normal*-hydrogen gas thermometer between 0 °C and 100 °C. The symbol °C that was used for this and the ITS-27 scale indicated degrees centigrade; the ITS-48 changed the name of the symbol to degrees Celsius (after the Swedish astronomer who was one of the two persons who independently proposed the centigrade scale, the scale based on the definition that the difference between the ice point and the boiling point of water was exactly 100 degrees). The International Temperature Scales that followed the NHS were based on fixed points with assigned temperature values based on measurements of the thermodynamic temperature, standard thermometers and interpolation equations. Until the ITS-27 was adopted in 1927, BS maintained NHS, adopted by the CIPM in 1887, using 16 *verre dur* mercury thermometers.

The purpose of the ITS-27 and of the subsequent International Temperature Scales has been well expressed in the introduction to the ITS-48 [4]:

“The experimental difficulties inherent in the measurement of temperature on the thermodynamic scale led to the adoption in 1927, by the Seventh General Conference of Weights and Measures, of a practical scale which was named the International Temperature Scale. This scale was intended to be as nearly identical with the thermodynamic centigrade scale as was possible with the knowledge then available. It was designed to be conveniently and accurately reproducible and to provide means for specifying any temperature on the International Scale within much narrower limits than was possible on the thermodynamic scale.”

**Fig. 1:**
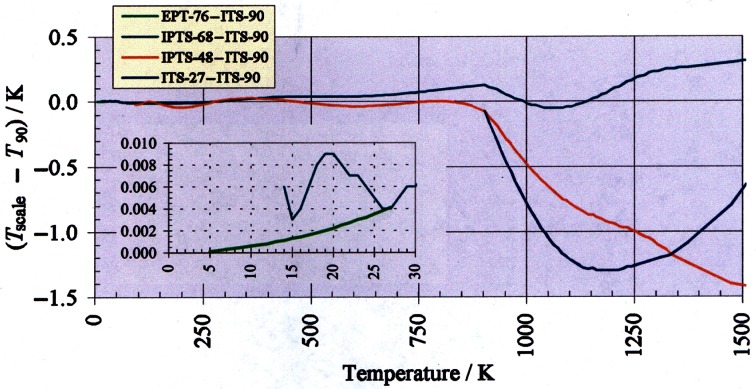
The differences between ITS-90 and EPT-76, IPTS-68, ITS-48, and ITS-27.

Figure 1 shows differences between the ITS-90 and the earlier EPT-76, IPTS-68, ITS-48, and ITS-27. The difference between ITS-90 and IPTS-68 reflects the more recent determination of the difference in the thermocouple range of the IPTS-68 (630.615 °C to 1064.18 °C) [12].

#### 3.1 International Temperature Scale of 1990 (ITS-90)

**Fig. 2:**
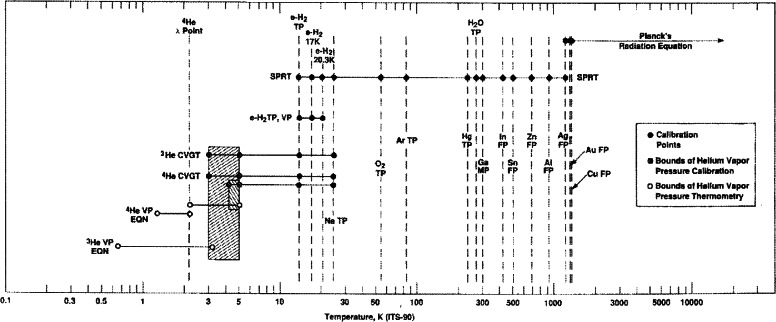
A schematic of the ITS-90 showing the temperatures of the defining fixed points (or phase equilibrium states) on the scale and the temperature ranges defined by interpolating instruments and equations. For assigned values of defining temperatures, see Table 1.

The ITS-90 extends upwards from 0.65 K to the highest temperature measurable by spectral radiation thermometry in terms of the Planck radiation law. The ITS-90 is defined in terms of 17 fixed points; vapor pressure/temperature relations of equilibrium-hydrogen (e-H_2_), ^4^He, and ^3^He; ^4^He or ^3^He constant-volume gas thermometers (CVGTs); standard platinum resistance thermometers (SPRTs); and radiation thermometers. The Pt-10 % Rh vs. Pt thermocouple that formerly defined the region from 630 °C to the Au freezing point (FP) has been replaced by high-temperature SPRTs (HTSPRTs). The spectral radiation thermometer can be referenced to either the Ag, Au, or Cu FP. Figure 2 is a schematic representation of the ITS-90 showing the defining fixed points and temperature ranges defined by interpolation thermometers and equations. The SPRT is the only contact-type interpolating instrument of the ITS-90 that directly disseminates the scale. In the previous International Temperature Scales, the standard Pt-Rh/Pt thermocouple served also in that position.

**Fig. 3:**
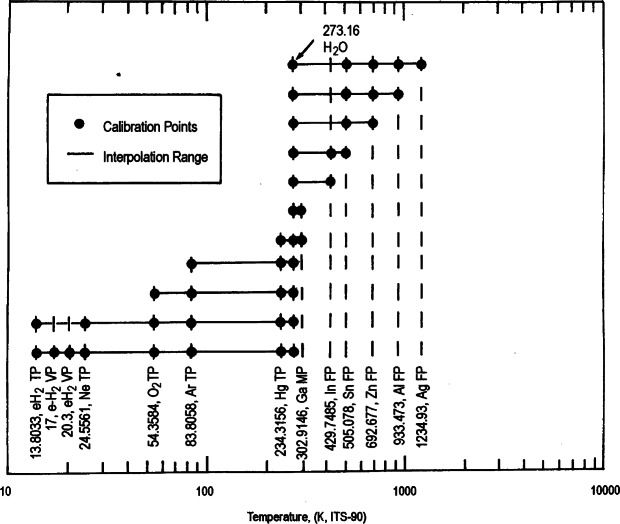
A schematic of the ITS-90 temperatures in the range specified for the platinum resistance thermometer, showing the various defined subranges and the temperatures of the defining fixed points required for calibration in the subrange.

The ITS-90 is designed with a number of ranges and subranges that overlap, giving different definitions of *T*_90_ that have equal status. The temperature differences that may arise are of negligible practical importance. Figure 3 shows the temperature range specified for SPRTs, with various defined subranges, and temperatures of the defining fixed points that are required for calibration.

##### 3.1.1 Realization of the ITS-90 at NIST

###### 3.1.1.1 Realization Below 84 K

Below 84 K, the ITS-90 has four different definitions: (1) ^3^He vapor-pressure thermometry (0.65 K to 3.2 K); (2) ^4^He vapor-pressure thermometry (1.25 K to 5.0 K); (3) interpolating constant-volume gas thermometry (3.0 K to 24.5561 K), with calibrations at a ^3^He or ^4^He vapor-pressure point between 3 K and 5 K, at the e-H_2_ triple point (TP) (13.8033 K), and at the Ne TP (24.5561 K); and (4) TPs over the range 13.8033 K to 83.8058 K, plus two additional temperatures close to 17.035 K and 20.27 K (determined either by using a gas thermometer or the specified temperature-vapor pressure relationship of equilibrium-hydrogen—See Table 1), at which capsule standard platinum resistance thermometers (CSPRTs) are calibrated and used for interpolation between the points. In order for a CSPRT to be used below 84 K, however, it must be calibrated also at the TPs of Hg and H_2_O.

Over certain temperature ranges, there is overlap between two or more definitions (see Fig. 2). All definitions are considered equally valid over their respective ranges, allowing the possibility of non-uniqueness in the ITS-90 in the overlap ranges [13].

**Fig. 4:**
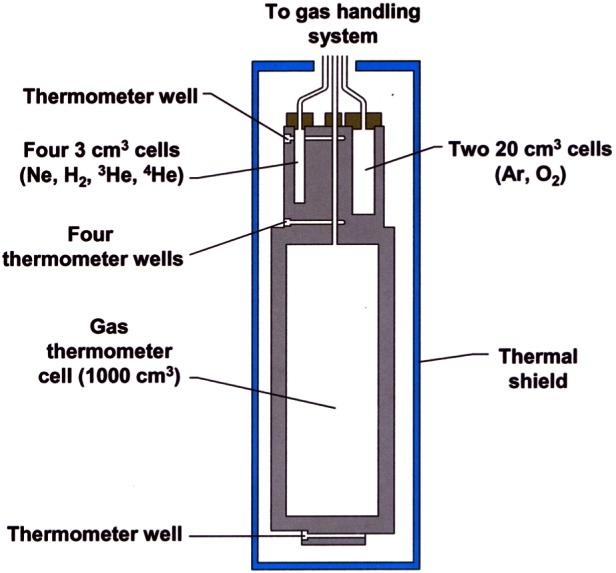
Schematic diagram of the copper block with ITS-90 realization cells.

In order to fully realize the ITS-90 below 84 K, NIST began construction of its Low Temperature ITS-90 Realization Facility (LTRF) in 1990. A brief description of the facility can be found in Ref. [14]. The LTRF was designed to realize the ITS-90 below 84 K using the guidelines published in *Guidelines for Realizing the International Temperature Scale of 1990 (ITS-90)* [15] and in *Supplementary Information for the International Temperature Scale of 1990* [16]. The centerpiece of the LTRF is a gold-plated cylindrical, oxygen-free high-conductivity (OFHC) copper block that contains seven sample cells for realizing the ITS-90 over this range (see Fig. 4). The largest is 1000 cm^3^ for the interpolating constant-volume gas thermometer (ICVGT). Four cells are used for realizing the triple points of Ar (20 cm^3^), O_2_ (20 cm^3^), Ne (3 cm^3^) and e-H_2_ (3 cm^3^). There is also a ^3^He vapor-pressure cell (3 cm^3^) and a ^4^He vapor-pressure cell (3 cm^3^). The e-H_2_ triple-point cell is used also for realizing the e-H_2_ vapor-pressure points at *T* ≈ 17.035 K and at *T* ≈ 20.27 K for calibrating CSPRTs. The e-H_2_ cell contains about 0.5 cm^3^ of ferric hydroxide powder, a catalyst for the conversion of orthohydrogen and para-hydrogen to their equilibrium distribution. The OFHC Cu block contains six thermometer wells, one at the top of the block, four at mid-height, and one at the bottom. The top and bottom wells can accommodate rhodium-iron resistance thermometers (RIRTs) and the mid-height wells can accommodate either CSPRTs or RIRTs. The resistances of the thermometers are measured with a commercial ac bridge using a standard resistor calibrated at NIST. The OFHC Cu block is surrounded by three copper shields in a vacuum space. The outer shield is immersed in an appropriate cryogenic liquid. Cooling of the copper block is accomplished by exchange gases for temperatures above 12 K and by a continuously recirculating ^3^He refrigerator for lower temperatures. Heating is performed with a resistive-wire heater wrapped around the Cu block.

**Fig. 5:**
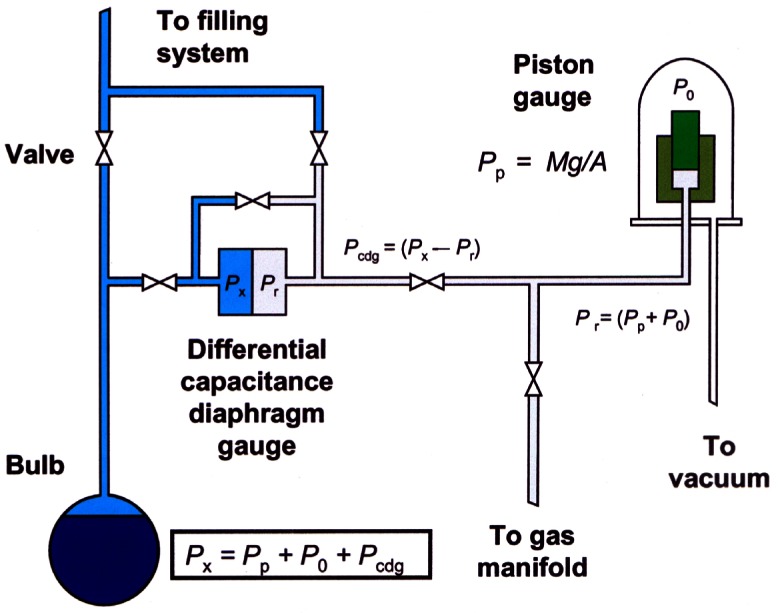
Pressure measurement system for the Low Temperature ITS-90 Realization Facility.

The ICVGT and vapor-pressure realizations require a pressure-measurement system (see Fig. 5), which is a combination of a piston gauge and a differential capacitance diaphragm gauge (DCDG). The piston gauge generates an accurately known pressure, *P*, and the DCDG measures the pressure difference between that of the cell and that generated by the piston gauge. The piston gauge pressure can be made to be very close to that of the cell, so that the pressure difference across the DCDG is small (< 20 Pa). With such a system, the relative standard uncertainty (*k* = 1) in the absolute pressure measurement is 12 × 10^−6^ and in the relative pressure measurement, it is 3 × 10^−6^. All cells requiring pressure measurement have individual DCDGs but use the same piston gauge.

A description of the triple-point realizations can be found in Ref. [17]. For these realizations, the Cu block is thermally isolated from the shields around it to make the heating of the block adiabatic. Before each melt, the Cu block is cooled to a temperature that is several kelvins below the triple-point temperature of the sample. It is then heated to a temperature that is slightly below the triple-point temperature and kept there for several hours to permit equilibration. Then the temperature is increased through the triple-point transition by successive constant increments of heat. After each increment of heat, the cell is allowed to come to thermal equilibrium. During this time, the temperature is monitored with one of the resistance thermometers. The size of the heat increments is typically 1/12 the heat-of-fusion. The period of time allowed for reaching thermal equilibrium after each heat increment is determined experimentally. At the end of the waiting period, the resistance *R* of the monitoring thermometer is measured. Data consisting of these final equilibrium resistance readings as a function of applied heat are used to determine the beginning of the melt, the end of the melt, and the heat-of-fusion. Subsequently, plots of thermometer resistance as a function of 1/*F*, where *F* is the fraction of material melted, are made. The final resistance is extrapolated to 1/*F* = 1 to provide the triple-point resistance R_TP_. At an appropriate point on the plateau of one of the melts, the resistances of all CSPRTs in the Cu block are measured. These resistances are corrected to correspond to 1/*F* = 1 by using the readings of the monitoring thermometer. Expanded uncertainties (*k* = 2) of realization for the triple points are 0.07 mK for Ar, 0.06 mK for O_2_, 0.21 mK for Ne, and 0.15 mK for e-H_2_.

Procedures for realizing the ITS-90 using the ICVGT are described in Ref. [18]. The ICVGT is filled with approximately 0.16 mol of ^4^He. The measurements with the ICVGT are at intervals of about 1 K. At each point, the temperature of the OFHC Cu block is brought to the selected temperature. The cryostat is allowed to equilibrate and then the resistances of the thermometers are measured. The pressure measurement is performed by first balancing the piston gauge and then measuring the pressure difference across the DCDG. Corrections to this pressure measurement are then made for the dead space between the ICVGT and the DCDG. Corrections are also made for the aerostatic pressure head and for the thermomolecular pressure difference. Measurements are made of gas pressures that correspond to the ITS-90 fixed points (5.0 K, 13.8033 K, and 24.5561 K) to calibrate the gas thermometer. The 5.0 K point involves the measurement of ^4^He vapor pressure. The latter two fixed points are triple-points of e-H_2_ and Ne, respectively. In practice, the three fixed-points are realized in the copper block first, and then the readings of the resistance thermometers are used to set the block temperature to the fixed-point temperatures to calibrate the gas thermometer. Once the ICVGT has been calibrated, the ITS-90 is realized with it by using the measured pressures and Eq. (4) of Ref. [1]. The RIRTs in the Cu block are then calibrated in terms of the ICVGT. The uncertainty (*k* = 2) of measurements with the ICVGT varies from 0.09 mK at 5 K to 0.26 mK at 24.5561 K.

The procedure used in the vapor-pressure/temperature measurements of ^3^He, ^4^He and e-H_2_ is described in Refs. [14,19]. For each vapor-pressure point, the Cu block is brought to the selected temperature and left to equilibrate. The resistances of the thermometers are then measured. The pressure is measured as described above for the ICVGT. Corrections to the measured pressure are made for the aerostatic pressure head and for the thermomolecular pressure difference. The ITS-90 temperature is then obtained from the measured pressure and Eqs. (6) or (11) of Ref. [1], and that value is assigned to the corresponding resistance of the thermometers. The uncertainties (*k* = 2) for the He vapor-pressure realizations are 0.1 mK or less over 97 % of the ranges of the ITS-90 definitions. In the lower 3 % of the ranges, the uncertainties increase to as high as 0.3 mK because of the increasing thermomolecular pressure correction. The uncertainties (*k* = 2) in the two e-H_2_ vapor pressures near 17.0 K and 20.3 K are 0.15 mK.

The LTRF was designed to calibrate in-house “reference-standard” resistance thermometers consisting of selected CSPRTs and RIRTs for NIST only. Customer thermometers are calibrated against these resistance thermometers in a comparator block located in a separate facility (see Sec. 6.1.2). Realization of the ITS-90 in the cryogenic range was completed in 1996, and since that year the scale below 84 K that is disseminated by NIST is traceable to the realization measurements made in the LTRF. NIST intends to realize the ITS-90 below 84 K in the LTRF to re-calibrate the reference-standard resistance thermometers at 5 year intervals to minimize scale uncertainties due to possible drifts of the thermometers.

The LTRF has been used also for studies of the scale, in particular the non-uniqueness of the ITS-90 [13] over the ranges of definition overlap. Results were published in 1996 [14] on the non-uniqueness over the range 1.25 K to 3.2 K, in which the scale is defined by the vapor-pressure/temperature relations of both ^3^He and ^4^He. The non-uniqueness over this range was found to vary between 0.1 mK and 0.3 mK. Results also were published [19] on non-uniqueness over the range 13.8033 K to 24.5561 K, in which the ITS-90 is defined by both ICVGTs and SPRTs. The maximum non-uniqueness found over this range was 1.55 mK, which occurs at 15 K.

To date, NIST is the only national laboratory to realize the ITS-90, as it is defined, from 0.65 K to 84 K in its entirety and it also is the only laboratory that has published determinations of the ITS-90 non-uniqueness below 25 K.

###### 3.1.1.2 Realization in the Range 83 K to 1235 K

The SPRT range from 83 K to 1235 K is defined by nine fixed points: Ag FP, Al FP, Zn FP, Sn FP, In FP, Ga melting point (MP), H_2_O TP, Hg TP and Ar TP. Samples of the highest purity are selected. Container materials were selected that would not contaminate the sample at the operating temperatures and are strong enough to endure multiple freezing and melting. Purified graphite was selected for Ag, Al, Zn, Sn and In; Teflon for Ga; borosilicate glass for H_2_O and Hg; and copper for Ar. Stainless steel is also used with Hg. The graphite container and its sample are protected from oxidation with an atmosphere of argon or helium gas. Table 2 lists the purity of the fixed-point substances, the container and holder materials, the amount of sample used, the immersion depth of the SPRTs in the thermometer well of the sample container, the controlled operating environment, and the uncertainties associated with the measurements using the fixed-point devices.

**Fig. 6:**
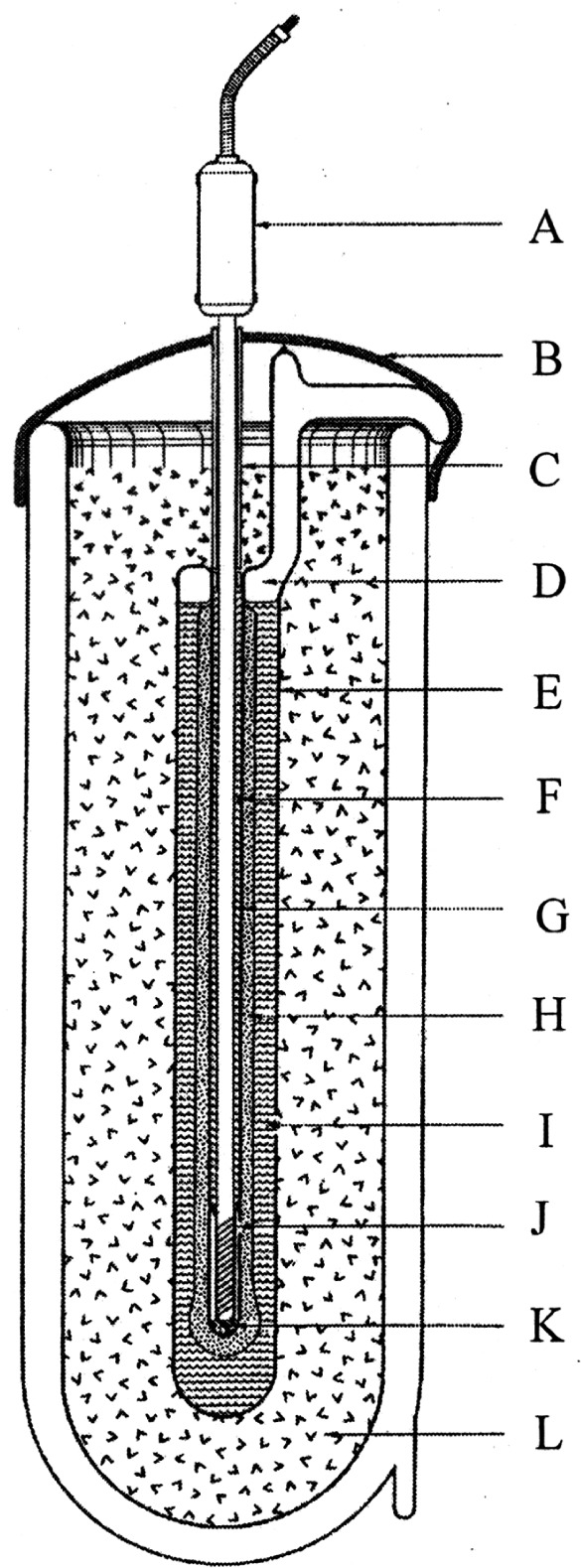
Water TP cell in an ice bath contained in a silvered Dewar. A—platinum resistance thermometer; B—heavy black felt shield against ambient radiation; C—polyethylene tube for guiding the SPRT into the thermometer well; D—water vapor; E—borosilicate glass cell; F—water from the ice bath; G—thermometer well (precision bore); H—ice mantle; I—air-free water; J—aluminum bushing with internal taper at upper end to guide the SPRT into the close-fitting inner bore; K—polyurethane sponge; L—finely divided ice and water.

The H_2_O TP is the most important fixed point of the ITS-90. The Kelvin Thermodynamic Temperature Scale (KTTS) is defined by assigning 273.16 K to the H_2_O TP, making the kelvin equal to 1/273.16 of the H_2_O TP temperature. All thermodynamic thermometry is referenced either directly or indirectly to this temperature. In the SPRT range, temperatures are determined in terms of the ratio of the observed resistance *R*(*T*_90_) at *T*_90_ to the resistance *R*(273.16 K) at the H_2_O TP, i.e., *W*(*T*_90_) = *R*(*T*_90_)/*R*(273.16 K), and the *resistance-ratio reference function*, which was designed to closely represent thermodynamic temperatures (see Refs. [1,20,21] for details). Figure 6 is a schematic of how a H_2_O TP cell of NBS design is used for calibrating an SPRT. For measurements with SPRTs at NIST, four H_2_O TP cells are maintained in a water bath held at 0.007 °C. For details of application and measurements at the H_2_O TP, see Ref. [22].

**Fig. 7:**
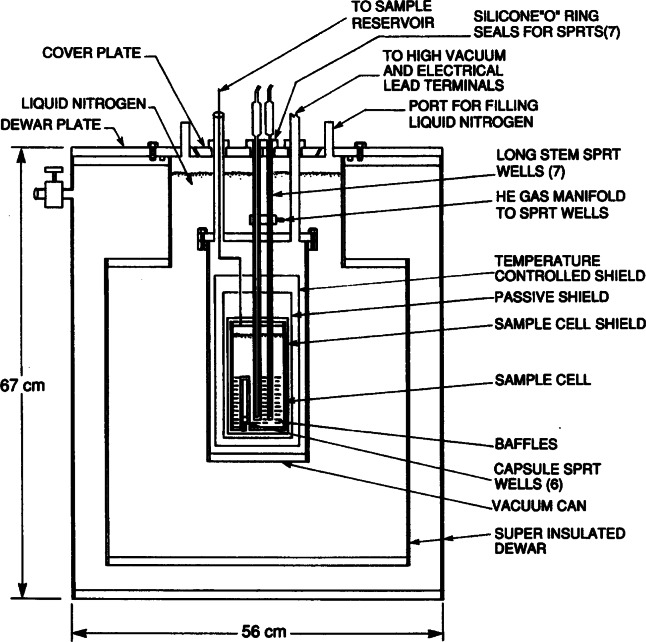
A schematic drawing of the argon triple-point apparatus for calibrating seven long-stem SPRTs and six capsule SPRTs. Six long-stem SPRTs surround a central SPRT well, which is sufficiently large to accommodate a holder for calibrating a capsule SPRT. At the bottom of the sample cell, six capsule SPRT wells are circularly arranged between the long-stem SPRT wells.

The Ar TP is realized by a method different from the others. The apparatus is operated immersed in liquid nitrogen. The outer vacuum jacket surrounds three sets of thermal radiation shields around the 300 cm^3^ copper sample cell, containing 15 mol of Ar, into which seven long, thin-wall stainless steel thermometer wells were inserted and soldered. During operation, the temperature of the tubes that extend above the sample cell is controlled close to the Ar TP temperature to temper the sheath of the SPRT. Figure 7 is a schematic of the apparatus for calibrating SPRTs at the Ar TP. The TP temperature realized with this apparatus agrees to within 0.1 mK with those Ar TPs obtained with sealed cells (see Refs. [23,24] and Sec. 5.1).

**Fig. 8:**
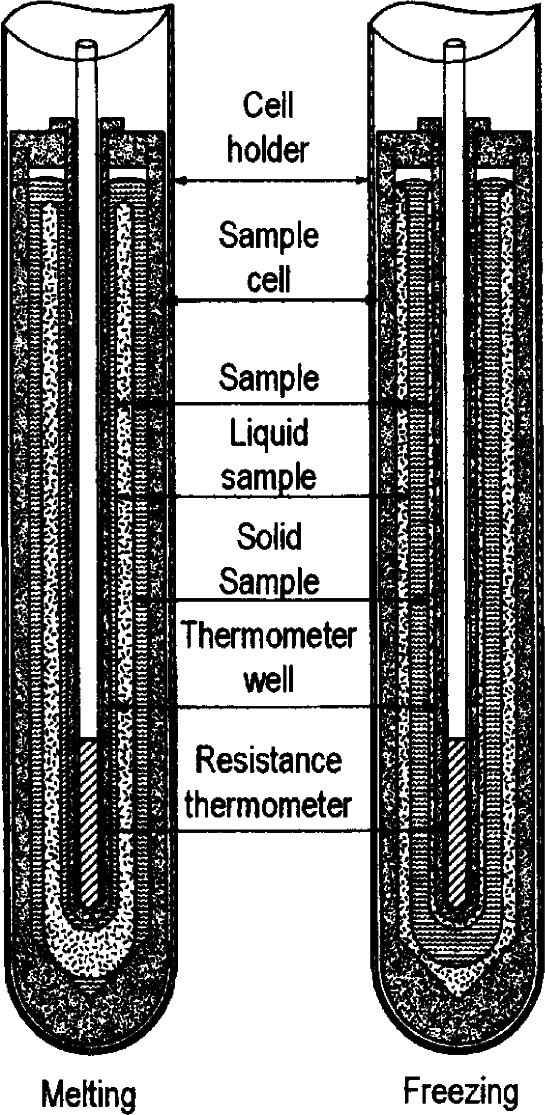
Idealized liquid/solid (L/S) equilibrium conditions inside fixed-point cells used in freezing and melting experiments. In freezing experiments, as the solid layer on the crucible wall thickens, its L/S interface approaches the L/S interface of the thin solid layer on the thermometer well. Similarly, in melting experiments, as melting advances, the outer L/S interface approaches the inner L/S interface.

In the development of fixed-point devices at NBS/NIST to achieve the best measurement accuracy in the calibration of SPRTs, attention has been given to having multiple phase-equilibrium surfaces to provide uniform surface temperatures for the SPRT. The H_2_O TP of Fig. 6 shows two equal-temperature equilibrium surfaces, one at the inner liquid-solid interface at the inner melt and the other at the outer surface of the mantle. Likewise, in the realization of the FP or the MP of metal fixed points, the operating procedure is designed to surround the SPRT in the sample container well by two equal-temperature equilibrium surfaces. Figure 8 is an idealized representation.

**Fig. 9:**
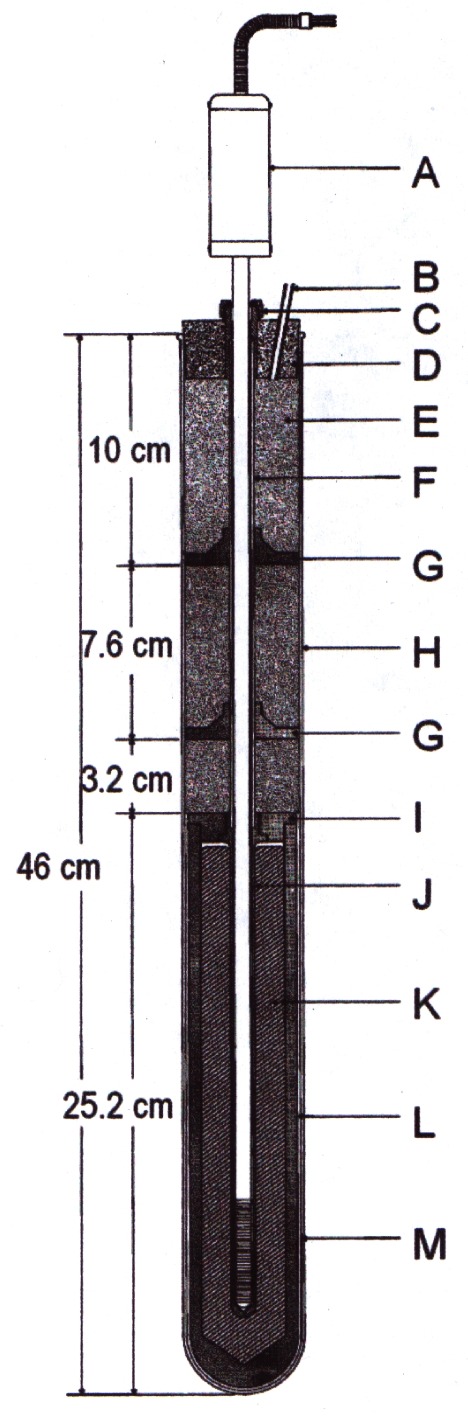
An SPRT in an indium, tin or zinc freezing-point cell. A—SPRT; B—to helium gas supply and pressure gauge; C—thermometer/helium gas seal with silicone rubber; D—silicone rubber stopper; E—thermal insulation (Fiberfrax); F—thermometer guide tube [precision bore tube, ground (matte finish) to uniform outside diameter]; G—heat shunt (graphite) in close contract with F and with H; H—borosilicate glass cell (holder) [precision bore tube, ground (matte finish) to uniform outside diameter]; I—graphite lid (cap) for the graphite crucible; J—graphite thermometer well; K—metal sample; L—graphite crucible; M—thermal insulation (Fiberfrax paper) between the graphite crucible and the borosilicate glass holder.

**Fig. 10:**
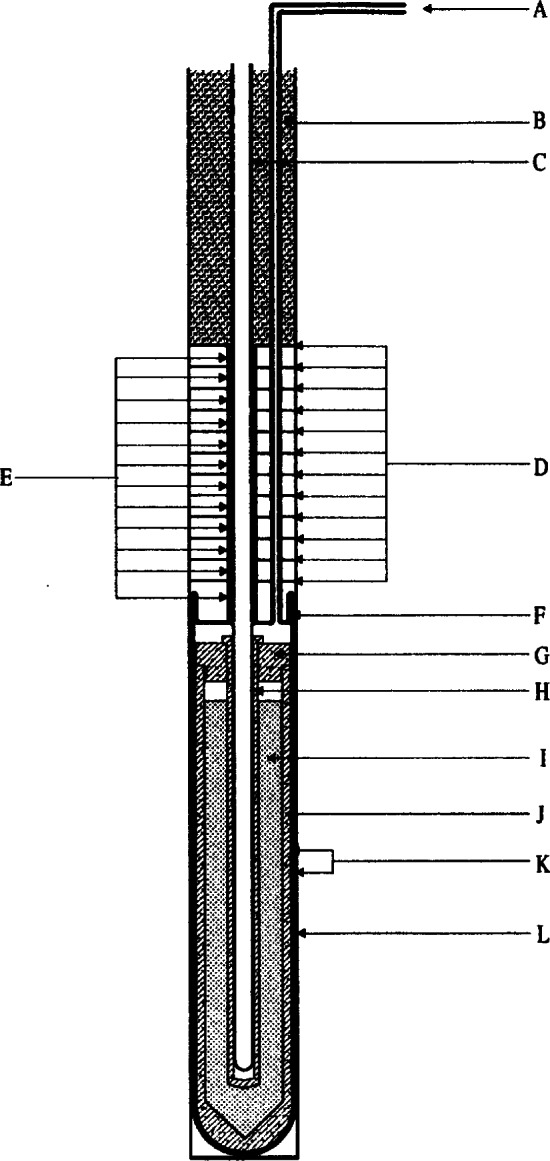
Aluminum or silver freezing-point cell. A—matte-finished silica-glass pumping tube; B—thermal insulation (Fiberfrax); C—matte-finished, silica glass thermometer guide tube; D—twelve Inconel radiation shields; E—thirteen silica-glass spacers; F—silica-glass envelope with a matte-finished, silica-glass re-entrant well; G—graphite cap for the graphite crucible; H—graphite re-entrant well; I—metal sample; J—graphite crucible; K—silica-glass tape for cushioning; L—Inconel protecting tube.

The wells for the long-stem SPRT are made sufficiently deep to eliminate “stem conduction.” The depth of the thermometer well of the container for the high-purity fixed-point substance is limited. To temper the SPRT sheath that extends above the sample container, the container is placed inside a long tubular “holder” that is inserted into a deep tube furnace or liquid bath operated at a temperature within 1 K of the FP or MP of the sample. A borosilicate glass holder is used with Zn FP, Sn FP and In FP graphite containers (see Fig. 9) and an Inconel metal holder is used with Al FP and Ag FP graphite containers (see Fig. 10). As an added protection, the graphite containers of Ag and Al are completely enclosed in silica glass before being placed inside Inconel metal holders. The sheath of the SPRT is tempered in the thermometer guide tube, which is centrally mounted above the thermometer well of the sample container. The guide tube is heated close to the furnace temperature by thermal bridges of graphite disks between the holder and the guide tube. In the cases of the holder for the Al FP and the Ag FP devices, twelve Inconel metal disk thermal radiation traps are mounted on the guide tube. Platinum disks, however, are preferred in order to eliminate the possibility of contamination of the thermometer. The stem conduction is considered eliminated when readings of the SPRT at different depths of immersion at the bottom 3 cm to 8 cm of the well (depending upon the SPRT and fixed-point device) correspond to the effect of the hydrostatic head (see Refs. [1,25]).

In the realization of the freezing point of Ag, Al, Zn, Sn or In for the calibration of (HT)SPRTs, the sample is melted overnight in the furnace held about 5 K above the freezing point. In the morning with a “check (HT)SPRT” in the thermometer well, the furnace temperature is reduced to initiate the freeze. When recalescence is observed, the furnace temperature is set to within 1 K below the freezing-point temperature. The check (HT)SPRT is removed and two cold silica-glass rods are successively inserted into the thermometer well for about 5 min each to form a thin layer of solid metal around the thermometer well. The cold check (HT)SPRT is then reinserted into the cell and the equilibrium temperature measurements are made. The reading should agree with previous freezing-point temperatures of the fixed-point device or devices of the same metal to within 0.1 mK. As shown by Fig. 8, two equal-temperature equilibrium interfaces are formed by the procedure. Usually for a given freeze for all of the metals except Ag, about six test (HT)SPRTs, that are first preheated close to the fixed-point temperature, are successively inserted into the fixed-point device and calibrated. For Ag, usually only one HTSPRT is calibrated per freeze. After measurements on the test (HT)SPRTs have been completed, the resistance of the check (HT)SPRT is read. The second reading of the check (HT)SPRT must agree with that of the first to within 0.1 mK; otherwise the calibrations are repeated. In the case of Sn, which supercools about 25 K, the Sn FP device is pulled out of the furnace to initiate the freeze. When recalescence is observed, the cell is reinserted into the furnace that is operating within 1 K below the freezing point.

In the case where temperatures are observed at melting conditions, e.g., the TP of Ga or Hg, the metal sample is frozen first; then the fixed-point device is inserted into a deep bath that is controlled about 1 K above the melting point. Next, a long heater is inserted into the thermometer well to form an inner melt. The bath liquid tempers the SPRT sheath that extends above the fixed-point device. Figure 8 shows the two equal-temperature equilibrium surfaces for melting experiments. For details of freezing and melting experiments with metal fixed-point cells, see Refs. [26–28]. Table 2 lists the total of Type A and Type B uncertainties, along with the expanded uncertainties *U* (*k* = 2) in measurements with each of the fixed-point devices. Type A uncertainties represent many measurements of check (HT)SPRTs that are associated with each of the fixed-point cells. Type B uncertainties reflect principally the effect of impurities in the fixed-point samples and the effect of physical and thermal geometry on the (HT)SPRT in the fixed-point device during measurements.

### 4. Thermodynamic Temperature Measurements at NBS/NIST

#### 4.1 Thermodynamic Temperature Measurements Utilizing Ideal Gases

The quantity that is termed temperature is well-defined by the laws of thermodynamics; measures of temperature that are defined to be consistent with the laws of thermodynamics are said to be thermodynamic temperatures. The measurement of thermodynamic temperature is based on a physical system that can be created in the laboratory and whose temperature is related to a set of measurable properties. The difference between temperature on the ITS-90, denoted *T*_90_, and the thermodynamic temperature, denoted *T*, can be determined by placing laboratory thermometers calibrated on the ITS-90 in the same apparatus that is used to determine *T*. Once the difference (*T* − *T*_90_) is known for a range of temperatures, this information can be used to improve future versions of the international temperature scale.

Early thermodynamic thermometers were based on the equation of state of an ideal gas, for which determinations of gas density and pressure enabled determination of the gas temperature. Significant experimental contributions by NBS began with the work of Hoge and Brickwedde [29], who calibrated an ensemble of resistance thermometers against a gas thermometer to establish a scale (known as the NBS-39 Scale) for the calibration of thermometers from 14 K to 83 K. At a later date, a program in gas thermometry at temperatures above 273 K was begun at NBS, as described in a review by Schooley [30] of gas thermometry work at NBS/NIST up to 1990. This research program culminated in the results of Guildner and Edsinger [31] from 273 K to 730 K and in the results of Edsinger and Schooley [32] from 503 K to 933 K using constant-volume gas thermometry. These results formed the basis of the ITS-90 from 373 K to 730 K, and also served as a reference point near 730 K for the radiometry work that defined the ITS-90 at higher temperatures. Unfortunately, the two sets of CVGT data differ by an amount equal to 12 mK at 500 K and rising to 30 mK at 730 K, which is much larger than the combined measurement uncertainty and which limits the thermodynamic accuracy of the ITS-90. The source of the discrepancy between the CVGT results has not been resolved.

An alternative to CVGT is the acoustic thermometer, which again relies on a simple relationship between thermodynamic temperature and measurable properties of the gas. The property to be measured in this case is the speed of sound *u* of a monatomic gas. Early measurements at NBS relied on an acoustic interferometry technique to measure thermodynamic temperatures from 2 K to 20 K [33]. To achieve higher accuracy, the value of *u* may be determined from measurements of the frequencies of acoustic resonances in a gas-filled spherical shell of volume *V*, a technique developed by Moldover and coworkers [34]. In the limit of zero gas density, kinetic theory and hydrodynamics give the dependence of *u* on *T*:

$$mu^{2}=\gamma kT,$$ (2)

where *m* is the mass of one molecule, *γ* is the specific heat ratio, and *k* is the Boltzmann constant. For monatomic gases *γ* = 5/3. Measurements of the frequencies of microwave resonances within the same shell determine the thermal expansion of the resonator cavity. The equation linking the measured frequencies to *T*, neglecting small corrections, is

$$\begin{array}{c} \frac{T}{T_{w}}={\left[\frac{u\left(T\right)}{u\left(T_{w}\right)}\right]}^{2}={\left[\frac{V\left(T\right)}{V\left(T_{w}\right)}\right]}^{2/3}{\left[\frac{f_{a}\left(T\right)}{f_{a}\left(T_{w}\right)}\right]}^{2}\\ ={\left[\frac{f_{m}\left(T_{w}\right)}{f_{m}\left(T\right)}\right]}^{2}{\left[\frac{f_{a}\left(T\right)}{f_{a}\left(T_{w}\right)}\right]}^{2}, \end{array}$$ (3)

where *T*_w_ is the triple point of water (273.16 K exactly) and *f*_a_ and *f*_m_ are the acoustic and microwave resonance frequencies.

**Fig. 11:**
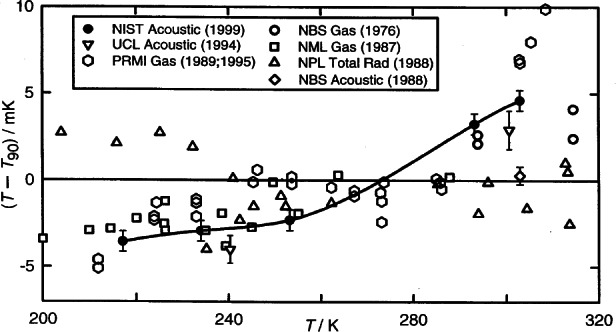
The difference between recent determinations of thermodynamic temperature and *T*_90_ in the range 200 K to 320 K. Citations can be found in Ref. [28].

As shown in Fig. 11, recent acoustic thermometry results at NIST [34] have determined thermodynamic temperature with a standard uncertainty of 0.6 mK in the temperature range 217 K to 303 K. The discrepancies of the CVGT work and the recent success at measuring thermodynamic temperatures near 270 K with an acoustic thermometer have motivated the development of an acoustic thermometer for determining the thermodynamic temperature above 500 K [35].

**Fig. 12:**
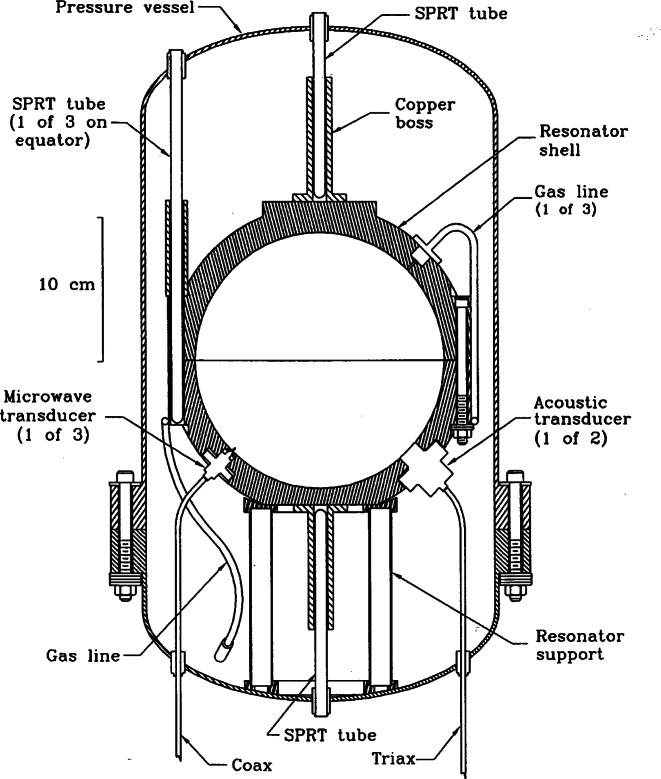
Simplified cross section of the NIST acoustic thermometer, showing the 3 L resonator, the pressure vessel, and associated plumbing and electrical connections. The furnace surrounding the pressure vessel is not shown.

Distinct advantages of acoustic thermometry over earlier CVGT work include higher precision, the ability to conduct experiments with continuously flowing gas to maintain purity, and the ability to use microwave resonances to characterize the volume of the resonator cavity *in situ*. The present NIST effort seeks to greatly expand the temperature range of precision acoustic thermometry and to benefit from the lessons learned while conducting the lower temperature measurements. The NIST acoustic thermometer, shown in Fig. 12, has the following features:

- Operation up to 800 K. Discrepancies between the NBS/NIST CVGT data become significant at temperatures above 500 K. Measurements at the zinc freezing point (692.677 K) are desirable, because the determined value of (*T* − *T*_90_) at the fixed-point temperature does not depend on the non-uniqueness of the SPRTs [13], which is a measure of the interpolation error between fixed points on the ITS-90.
- Continuous purging of the resonator cavity. Contamination of the gas in the resonator is proportional to its residence time, or inversely proportional to flow rate. Continuous purging reduces gas residence time approximately two orders of magnitude relative to the residence time in CVGT experiments. Sensitive pressure control techniques are used to limit adiabatic temperature variations in the gas, caused by pressure fluctuations, to 0.5 mK or less.
- Direct measurement of impurities in the gas exiting the resonator. A gas chromatography system can detect impurities in the sample gas with a mole fraction sensitivity better than 0.5 × 10^−6^.
- Simultaneous microwave and acoustic measurements. At elevated temperatures, creep of the spherical shell is a significant possibility. Microwave measurements that are concurrent with the acoustic measurements are used to correct for creep at each datum point. For the acoustic measurements, novel capacitance transducers have been developed that utilize a monocrystalline silicon diaphragm and alumina insulators, enabling operation at temperatures up to 800 K.
- Stable and inert materials. We use no elastomers, which have been a significant source of outgassing in previous acoustic thermometers. The materials exposed to high temperatures include stainless steel, copper, alumina, platinum, and gold.
- Well-characterized resonator temperature. Up to five long-stem SPRTs, calibrated on the ITS-90, may be used to measure the resonator shell temperature. To minimize temperature fluctuations and spatial variations, the pressure vessel is encased in three concentric aluminum shells that are actively temperature controlled, and the thermal couplings between the aluminum shells, the SPRTs, and the spherical resonator have been carefully modeled.
- A resonator cavity of approximately 3 L. Previous measurements with resonators of at least this volume agree well with theoretical predictions of the acoustical losses.

This acoustic thermometer has been fabricated and successfully tested up to 500 K. Work continues with a goal of measuring (*T* − *T*_90_) over the range 273 K to 800 K with a standard uncertainty not exceeding 0.6 mK near 273 K and 3 mK at 800 K. These measurements, we hope, will contribute to significant improvements in the thermodynamic accuracy of the next international temperature scale.

#### 4.2 Thermodynamic Temperature Measurements Utilizing Johnson Noise

The random fluctuations in current and voltage in a normal conductor, generally known as “Johnson noise” [36], are a result of the thermally activated motion of the conduction band electrons. Consequently, as first shown by Nyquist [37], the mean square noise voltage <*V*^2^> across a resistance *R* in a frequency band Δ*f* is directly proportional to its absolute temperature *T*, in the low frequency-high temperature limit (*hf* << *kT*), or

$$\langle V^{2}\rangle =4kRT\Delta f,$$ (4)

where *k* is the Boltzmann constant. Numerous applications of Johnson noise thermometry (JNT) utilizing the Nyquist relation have been developed in the last 50 years since the 1949 publication by Garrison and Lawson [38] describing the first practical instrument. The significance of this work was recognized early on by the NBS staff. In particular, Hogue [39] was the first to critically examine the limitations inherent in the measurement technique utilized by Garrison and Lawson. The subtleties of amplifier gain and noise level being dependent on source impedance, as described by Hogue, were subsequently taken into account in later JNT designs.

Many of these early efforts are described in the review article by Kamper [40].

Kamper and Zimmermann [41], working at the NBS Boulder Laboratories, were also the first to apply the high sensitivity inherent in the Josephson effect to measuring temperatures in the range of 4 K and below. Soulen [42] later refined this technique into a special type of JNT instrument known as an “R-SQUID,” which was used to establish thermodynamic temperature between 520 mK and 6.5 mK.

Despite the great technological advances during the last few decades, the general measurement problems of JNT have remained highly challenging due to the extraordinarily small signal level, which is only about 1.26 nV/√Hz for 100 Ω at 273 K. Until recently, the benchmark for accuracy in practically all JNT instruments was 0.1 %. This fact has relegated JNT as a thermodynamic technique to the fringes of contact thermometry (i.e., *T* < 1 K or *T* > 1000 K), where the generally more accurate gas-based techniques are not practical. At the same time, some specialized industrial applications of JNT have been developed [43] which take advantage of the primary thermometer status of JNTs in order to solve difficult calibration problems in high-temperature and highly-ionizing-radiation environments. For these applications, such as in nuclear and fossil fuel reactor environments, an uncertainty of 0.1 % is very competitive with all other types of industrial contact thermometers available [e.g., platinum resistance temperature detectors (RTDs) and base metal thermocouples].

Recently, increasing amounts of technical sophistication and digital processing techniques have been brought to bear on the JNT problem [44]. As a result, it is now possible for a JNT system to achieve relative uncertainties, using switched-input noise-correlation techniques, which are smaller than 0.01 % over a broad range of temperatures [45]. The significance of these advances, originating at the Forschungzentrum Jülich in Germany, has been recognized by various national metrology laboratories in Europe as well as by the staff at NIST. A European collaboration between the researchers at the Netherlands Measurement Institute (NMi), the Physikalisch-Technische Bundesanstalt (PTB), and the Forschungzentrum Jülich has recently demonstrated thermodynamic fixed-point determinations using a Jülich designed JNT system with relative uncertainties of (5 to 7) × 10^−5^ [45] at the Ga MP, Zn FP, Ag FP, and Pd FP.

Starting in late 1999, NIST initiated a program in JNT designed to advance the state-of-the-art using the recent advances in digital synthesis and signal processing techniques, together with advances in the Josephson pulse-Quantized Voltage Source (JQVS) [46]. The goal of the project is to create a JNT measurement system capable of achieving relative uncertainties of 1 × 10^−5^ in the range of temperatures between 83.8 K and 430 K. In addition, NIST will explore the potential for industrial level applications of this technology in those extreme and/or remote environments where the temperature must be accurately known over long periods of time without access to either fixed points or replacement of probes.

### 5. Device-Based Research

#### 5.1 Gas-Based Cryogenic Fixed Points

The triple points of certain chemically-pure elements and compounds, when realized via the sealed-cell technique, produce compact, transportable fixed-point standards in the range between 13.8 K and 216.6 K. These substances are gases at standard temperature and pressure (273.15 K, 101.325 kPa) and realizations of their triple points require cryogenic techniques. Sealed-cell techniques are well suited for the realization of four of the defining fixed points of the ITS-90 [1]: Ar (83.8058 K), O_2_ (54.3584 K), Ne (24.5561 K) and e-H_2_ (13.8033 K). In addition, the triple points of several other substances such as e-D_2_, N_2_, Kr, Xe, and CO_2_, while not defining fixed points on the ITS-90, are potentially useful for temperature scale research [47], e.g., the non-uniqueness of portions of the ITS-90 [13]. These fixed points are useful also for international scale comparisons [48], scale maintenance, and dissemination.

The inherent stability of the triple point results from all three phases of the sample being in thermal equilibrium. When a pure material of fixed amount attains the triple-point temperature, there are no remaining degrees of freedom in which the three phases may coexist. Heat may be absorbed or emitted by the sample undergoing melting or freezing under its own saturated vapor without a change in temperature. The latent heat of fusion that accompanies the first order phase transition provides a stable plateau in temperature, useful for calibrating thermometers.

Previous work at NBS/NIST has included realizations of the triple points of Ar [24], O_2_ [49], Xe [50], and Ne [51] using sealed cells of various designs. The fundamental theory and conventional practice of sealed cells has recently been reviewed by Pavese [52]. The generic sealed cell consists of a permanently sealed pressure vessel with a ballast volume; a sample volume for the condensed portion of the sample; a thermometer well insert; a heat exchanger; and a heating element. In the NBS/NIST sealed-cell designs discussed here, the volume of the pressure vessel is primarily ballast, ranging from 20 cm^3^ to 50 cm^3^, and the cells contain the pressure of the room temperature gas. Storage pressures need not exceed 12 MPa at 300 K for cells of this size, which hold samples of 0.2 mol or less.

The thermometer well inserts are large enough to accommodate three capsule-type thermometers, either CSPRTs or RIRTs. The insert exchanges heat with the solid and liquid phases of the sample by confining the condensed sample to form an annular mantle surrounding the thermometer well insert. The heat exchange surface is optimized between the competing requirements of maximum surface area and minimum flow impedance in the annular sample space. In the latest NIST designs, this is accomplished through a double helical groove geometry.

Current capabilities at NIST related to sealed cells include two all-metal gas handling manifold systems; a cryostat adapted for adiabatic measurements of melting plateaus using sealed cells; and a variety of cells made from type 316L stainless steel and oxygen-free copper. The gas manifold systems include one general purpose manifold, GM-1, suitable for any of the gases mentioned above except for H_2_ and D_2_. The GM-1 can fill cells in either gas phase or condensed phase and includes a high-temperature vacuum bake-out furnace for service up to 450 °C. The other gas manifold, GM-2, is a special system designed for H_2_ service using only condensed phase filling. The cryostat has an operating temperature range sufficient to realize all the triple points mentioned here and a sufficiently large sample space to accommodate up to three sealed cells at once. The cells currently being used at NIST are suitable for any of the above gases, with the exception of D_2_, which requires special materials and considerations (see below).

The current sealed-cell research and development efforts at NIST are focused on the production of chemically-pure H_2_ samples of mass fraction 99.9999 % using conventional spin-exchange catalysts of alpha ferric hydroxide. A related research topic at NIST concerns the analysis of the actual isotopic purity of prepared H_2_ samples relative to the deuterium to hydrogen (D/H) ratio of 156 μmol/mol, derived from Standard Mean Ocean Water (SMOW), as specified by the ITS-90. Relative isotopic abundance in e-H_2_ is a source of uncertainty in the ITS-90 due to the high sample to sample variation in the D/H ratio (e.g., 40 μmol/mol to 125 μmol/mol) of commercial gas bottles of high chemical purity H_2_. These variations are due to the different methods of synthesis employed commercially and the commensurate variations in the relative depletion of the heavier isotope with respect to an equivalent SMOW composition.

NIST is a participant in an international comparison of sealed triple-point cells ongoing at the PTB. As of this writing, NIST sealed cells of Ar, O_2_, and Ne have been compared with other cells at PTB, and there are plans to include an e-H_2_ cell. This comparison was originally conceived as an EUROMET project, but later it was expanded to include some non-EU countries.

Another active area of sealed-cell research is a collaboration with the Istituto di Metrologia “G. Colonnetti” (IMGC) to disseminate 0.05 mol samples of D_2_ with mass fraction 99.998 %. This D_2_ gas was originally prepared in 1986 [53] through a special process developed at the U.S. Department of Energy’s Mound Laboratory in Miamisburg, OH, which was designed to minimize contamination by the lighter isotope. The IMGC is transferring some of this gas from a storage cylinder into a number of sealed cells of different design for international dissemination, including cells to be used at NIST. Isotopically-pure deuterium is particularly challenging due to the presence of HD impurities from H_2_ contaminant gas in the nominal iron hydroxide catalysts as well as in the stainless steel cells themselves. Consequently, one is forced to use relatively weaker catalysts such as Gd_2_O_3_ which contain no water of hydration. In addition, special cell construction materials such as reinforced oxygen-free copper or vacuum-arc re-melt stainless steel are necessary to avoid H_2_ contamination of the D_2_. The long-term viability of deuterium sealed cells for triple-point standards, as prepared and stored with these considerations in mind, has not yet been conclusively determined.

#### 5.2 (Standard) Platinum Resistance Thermometer [(S)PRT]

In the investigations in 1881 by Callendar and in 1909 at BS, PRTs wound on mica crosses were used to measure the freezing-point temperatures of metals up to 1100 °C [54,55]. The reproducibility was on the order of 0.1 °C to 0.3 °C. The stability was dependent on the purity of the platinum wire and how well the platinum wire was protected from contamination by its supports and surroundings. Since that time, developments in platinum resistance thermometry have resulted in many improvements: higher purity of the Pt wire; smaller size of the Pt resistance element; supports for the Pt resistance coils that are nearly free of contamination and that maintain the Pt resistance coil in a nearly strain-free state; and increased accuracy of resistance measurements and of representation of the thermodynamic temperatures.

**Fig. 13:**
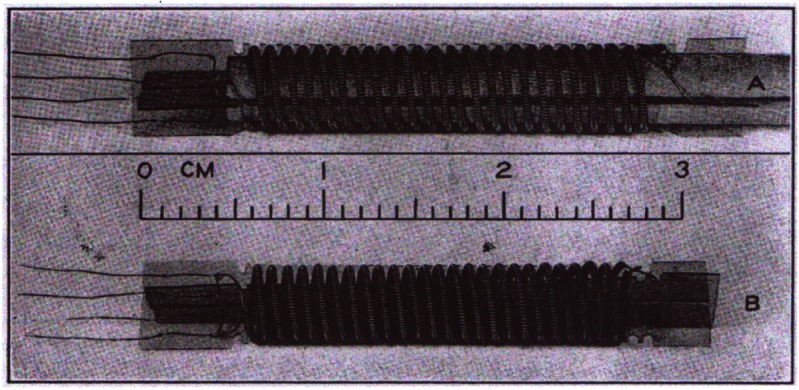
Large model of Meyers’ thermometer coil. A—mounted on mandrel; B—removed from mandrel (from Ref. [50]).

In 1932, Meyers of NBS described the design of an SPRT element consisting of a small helical Pt coil that was 5 mm in diameter and 32 mm in length and that was wound in a strain-free manner on a notched ruby mica cross [56]. Figure 13 is a photograph of the SPRT element. The size is comparable to that of most mercury thermometers. The element is mounted inside borosilicate or silica-glass tubes for long-stem SPRTs or is inserted into Pt tubes for CSPRTs. Both SPRTs have been commercially available since that time. The work of McLaren on reducing light transmission (piping) in glass sheathed SPRTs [57] and on eliminating external illumination of SPRTs [58], and the work of Berry on the thermal strain [59] and oxidation effects [60] in SPRTs have contributed much to achieving greater accuracy with SPRTs. Platinum resistance elements of other coil forms have been introduced, but SPRTs of Meyers’ design seem to give the best reproducibility below about 600 °C.

Investigations have been conducted at NBS and in other national laboratories to extend the SPRT scale to the Au FP [61–63]. The electrical resistivity of insulation supports is less at high temperatures. High-temperature SPRTs of 25.5 Ω, 2.5 Ω, and 0.25 Ω of several designs have been made and tested for the effects of insulation leakage [64,65]. On prolonged exposure to high temperatures, the Pt wire became susceptible to mechanical and thermal shock [66]. In some cases, grain boundaries were visible [67]. The removal of strains that were introduced during the manufacture of the Pt wire and in winding the Pt coil requires prolonged heating at high temperatures. Slow cooling of the HTSPRT from high temperatures is required to avoid freezing-in high-temperature lattice vacancies.

Oxygen is added to the heat exchange gas inside the SPRT sheath to maintain any metal impurities that might be present in the oxidized state. Free metals will alloy with Pt, especially at high temperatures. The oxygen also oxidizes the Pt, forming an oxide that has greater resistivity than Pt. Thus, when the SPRT coil is oxidized, its resistance is greater than when it is less oxidized. The error in the resistance ratio is small or negligible when the degree of oxidation is the same for the two resistance measurements that are required. The rate of oxidation seems to be the greatest in the range 300 °C to 400 °C [68]. With SPRTs filled to about one third of an atmosphere of dry air as an exchange gas, the Pt oxide is decomposed at about 500 °C. Slow cooling of HTSPRTs to about 500 °C and quickly cooling to the ambient temperature and then to the H_2_O TP should yield an accurate resistance ratio for the high temperature observation. See Table 2 for uncertainties (Type A) of measurements that can be achieved with SPRTs and HTSPRTs in different fixed-point cells up to the Ag FP.

#### 5.3 Thermocouple Thermometry

Historically, much of the research at NBS and NIST in thermocouple thermometry has focused on the determination of reference functions for a variety of thermocouple types. A thermocouple reference function, giving thermoelectric emf as a function of temperature, serves two purposes: it is a standard that thermocouples are manufactured to match, to within a specified tolerance, and it is a tool for calibration of thermocouples. With an accurate reference function, a thermocouple may be calibrated at only a small set of temperature values, and the thermoelectric emf at intermediate temperatures may be obtained between these values by first interpolating the deviation of the emf from the reference function, and then adding the deviation to the reference function value.

Each of the reference functions for the letter-designated thermocouple types are based in part on research performed at NBS. Major NBS contributions include (see citations in Ref. [69])

1. establishment of the first reference functions for types E, K, and N thermocouples;
2. improvement of the reference functions for types B, R, S, J, and T thermocouples; and
3. determination of reference functions for all of the base metal thermocouple types (E, J, K, N, and T) from 0 °C to temperatures as low as −270 °C.

All of the internationally-standardized and letter-designated thermocouple types have been adjusted to the ITS-90 temperature scale by NIST researchers and are now disseminated both in NIST publications [69] and in national [70] and international standards [71].

In addition to the work on reference functions, NBS/NIST researchers have made significant contributions to the development of calibration and fabrication techniques for high-temperature tungsten-rhenium alloy thermocouples [72], and in characterization of the drift of thermocouple emf values at elevated temperatures [73].

Although thermocouple thermometers are exceedingly simple in construction and have been in use for over a century, recent work at NIST [74–76], stimulated by publications of McLaren and Murdock [77], has documented the fabrication and use of thermocouples with uncertainties an order of magnitude better than previous reference standard thermocouples. Alloy thermocouples are limited in performance because oxidation or vaporization of one of the alloy components at high temperature alters the thermoelectric properties. Thermocouples fabricated from pure elements, either gold vs platinum (Au/Pt) or platinum vs palladium (Pt/Pd), do not suffer from preferential oxidation or vaporization. With careful annealing to place the thermoelements into a homogeneous and well-controlled physical state, and with careful measurements of the emf, expanded uncertainties (*k* = 2) as small as 10 mK at 960 °C are attainable.

**Fig. 14:**
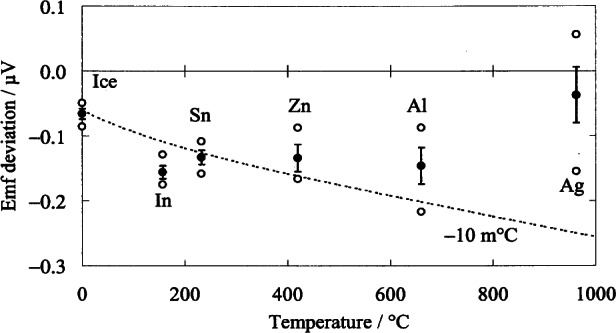
Deviation of emf values at fixed points of the SRM 1749 Au/Pt thermocouples from the NIST reference function. Full circle: average of 18 thermocouples; open circle: maximum and minimum values. The uncertainty bars indicate ± 1 standard deviation.

A set of Au/Pt thermocouples, available from NIST as Standard Reference Material 1749, were recently fabricated and calibrated at NIST. The calibration results, expressed as deviations from the NIST reference function, are shown in Fig. 14. The variations of emf values between the different thermocouples do not exceed the equivalent of 8.5 mK, an indication both of the reproducibility of the annealed state of the thermocouples and of the uniformity of the commercially-available gold and platinum wire used in their construction. For each thermocouple, the deviation of emf from the reference function can be accurately modeled by a quadratic function. The expanded uncertainty (*k* = 2) of this set of thermocouples is the equivalent of 8 mK from 0 °C to 962 °C, and then rising to 14 mK at 1000 °C. In comparison, a platinum-rhodium thermocouple can be calibrated to an expanded uncertainty not less than 0.1 K.

**Fig. 15:**
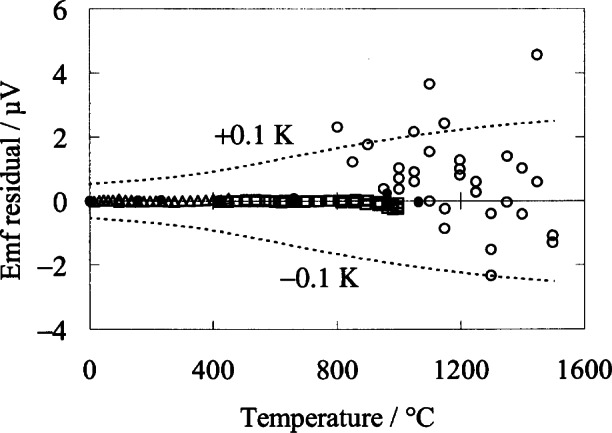
Residuals of data from a spline polynomial that forms the basis for the NIST/IMGC reference function for Pt/Pd thermocouples. Open triangle: SPRT comparison; open square: Au/Pt TC comparison; open circle: IMGC radiometry; full circle: fixed points.

Au/Pt thermocouples are the most accurate thermocouples available, but the melting point of gold at 1064 °C does not allow use at temperatures exceeding 1000 °C. Pt/Pd thermocouples have uncertainties approaching those of Au/Pt thermocouples, and have a maximum usage temperature of 1500 °C. A recent collaboration between NIST and IMGC (Italy) led to the development of a reference function [76] for Pt/Pd thermocouples for the temperature range 0 °C to 1500 °C, with expanded uncertainties not exceeding the equivalent of 11 mK up to 1050 °C, and rising smoothly to 0.3 °C at 1500 °C. Figure 15 shows residuals of the data from a spline function that forms the basis of the reference function. Up to 1064 °C, the data were obtained from measurements of the Pt/Pd thermocouples in fixed-point cells, and by comparison against SPRTs in stirred-liquid baths and against Au/Pt thermocouples in a copper isothermal block. From 800 °C to 1500 °C, the data were obtained by comparison measurements of the Pt/Pd thermocouples against a radiometer, which was calibrated on the ITS-90.

Commercialization of pure element thermocouples has been successful, but recommended procedures still need to be developed and disseminated for optimal use of these thermocouples in standards laboratories or in such demanding environments as semiconductor processing. Future work at NIST will be in this direction.

Another active area of thermocouple research is the development and application of thin-film thermocouples (TFTCs). As a consequence of the sub-micrometer thickness of TFTCs, these sensors have a very fast response time and do not thermally perturb the object being measured. Projects on TFTCs have included transparent TFTCs [78]; corrosion resistant TFTCs [79]; high-temperature metal silicide TFTCs [80]; and high-output intermetallic TFTCs [81]. NIST work has also pioneered improved methods for calibrating TFTCs [82], bonding of TFTCs to oxides, and calibrating radiometers [83]. The development of a thin-film/wire thermocouple wafer for calibrating light-pipe radiation thermometers is an ongoing project but it has already achieved smaller uncertainties (standard uncertainty of 2.1 °C at 900 °C) than any currently-existing commercial technology [84].

### 6. Maintenance and Dissemination of Temperature Scales

#### 6.1 Maintenance and Dissemination of the ITS-90 and Other Scales Below 84 K

##### 6.1.1 Prior Scales

The capability for performing calibrations of thermometers in the cryogenic range (i.e., *T* < 120 K) has been maintained at NBS/NIST since 1939 starting with the National Bureau of Standards Constant-Volume Gas Thermometer Scale of 1939 (NBS-39 Scale) [29]. Later, scientific and technical refinements, both within NBS/NIST and internationally, were carried over into the dissemination of the following scales in the cryogenic range: the National Bureau of Standards 1955 Scale (NBS-55) [85]; the National Bureau of Standards Provisional Temperature Scale 2–20 (NBS P2-20) [33]; IPTS-68 [86]; EPT-76 [87]; and finally the ITS-90 [88]. All of these scales were either laboratory thermodynamic scales or international practical scales that were generally too complicated to be realized outside of the national laboratory environment. These complications necessitated the use of reference thermometers with which to maintain these scales for calibration purposes. Such an approach then requires the use of a system of scale maintenance that periodically checks the references against known fixed points, and compares the reference thermometers amongst themselves. The measurement system for comparison then also serves as the means of providing comparison calibrations for customers’ thermometers, which has been the customary approach at NBS/NIST.

##### 6.1.2 The ITS-90

**Fig. 16:**
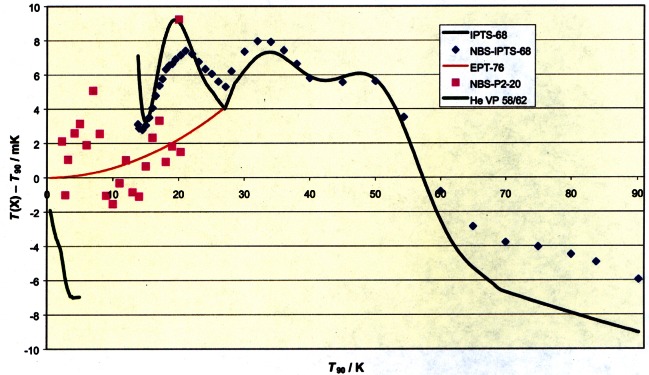
The difference in mK between various historical temperature scales in the cryogenic range and the ITS-90 as realized by NIST. The IPTS-68 curve represents the version as disseminated from the National Physical Laboratory (UK) (NPL), a different version was disseminated from the NBS (NBS-IPTS-68). The 1958 and 1962 He vapor-pressure scales (VP 58/62) were based on a vapor pressure relation for ^4^He and ^3^He. The NBS P2-20 scale was a provisional scale based on acoustic gas thermometry from 2 K to 20 K. The EPT-76 was another provisional scale based on paramagnetic susceptibility.

In the case of the ITS-90, the inherent complications in its realization below 24.5561 K prevented any national laboratory from completing a full realization, according to the definitions, before 1996. In fact, the only laboratory to do so even by the year 2000 is NIST [89]. As a consequence, between January 1990 and October 1996 the ITS-90 below 83.8 K was disseminated from NIST by a “wire scale” approximation [90], usually referred to as “ITS-90W” with temperatures denoted *T*_90W_. From October 1996 onward, temperatures *T*_90_ of the “as defined” ITS-90 below 83.8 K were disseminated [91], as a result of the completed NIST realizations of the ITS-90 from 0.65 K to 83.8058 K [14,18]. This scale change shifted the disseminated temperatures by less than 1 mK over this range. The ITS-90 sub-ranges at temperatures at or above 83.8058 K, as disseminated from NIST, continued according to definition during this time and were unchanged by this lower temperature scale shift. Figure 16 is a summary of all previous temperature scales disseminated from NBS/NIST since approximately 1965 over the range 0.5 K to 90 K.

Between 1972 and 1992, an ultra-low temperature scale below 1 K was developed at NBS/NIST. This scale was derived from thermodynamic temperature determinations using a SQUID-based Johnson noise thermometer [92], ^60^Co *γ*-ray anisotropy, and paramagnetic salt susceptibility [93]. These thermodynamic, or nearly thermodynamic, measurements were used to derive a ^3^He melting curve relation for the melting pressure *p*_m_ and temperature *T*, over the range 0.006 K to 0.7 K [94]. The difference between a temperature *T* on this scale and the NIST realization of the ITS-90 at *T*_90_ = 0.65 K is approximately 1 mK. Calibrations from NIST on this scale are no longer available.

The current NIST calibration capabilities in the cryogenic range cover most types of cryogenic resistance thermometers, including all types of capsule SPRTs for temperatures from 13.8 K and higher and RIRTs over temperatures between 0.65 K to 83.8 K. The calibrations in this range are performed in a recirculating ^3^He cryostat via an OFHC copper comparison block in vacuum. All low temperature comparison calibrations are arranged according to batches with no more than two such batch calibrations being scheduled per year.

Resistance thermometers made of rhodium with 0.5 % iron, known as RIRTs, were first developed by Rusby [95] in 1975 and are now available commercially. Because of their high sensitivity and stability for *T* < 24 K, NBS/NIST began using RIRTs in 1976 as reference thermometers for its EPT-76 over its range [10,87]. Stability tests on the RIRTs used at NIST were performed by Pfeiffer [96], who determined the differences between the temperatures indicated by the NIST reference RIRTs in 1982 and in 1990. He determined that the two RIRTs had undergone a maximum relative drift of 0.15 mK over that 8 year period. After NIST realized the ITS-90 below 24 K in 1996, it has calibrated customer thermometers using reference RIRTs that have been calibrated in its LTRF. Measurements of RIRT resistances are made with a commercial ac bridge, typically using currents of 0.2 mA and 0.283 mA for *T* > 1 K and 0.1414 mA and 0.2 mA for *T* < 1 K. Calibrations are made at 1 K intervals, and an 11th order polynomial series is fitted to the results. In 1999, NIST participated in the CCT Key Comparison 1, which is comparing various national laboratories’ realizations of the ITS-90; for these comparisons the NIST realization was represented by RIRTs calibrated in the NIST LTRF.

The ITS-90 in this range is maintained at NIST on a set of highly stable reference SPRTs and RIRTs. The reference RIRTs have been calibrated on the ITS-90 using the following defined sub-ranges: the ^3^He vapor pressure scale from 0.65 K to 2.0 K; the ^4^He vapor pressure scale from 2.0 K to 5.0 K; and the ICVGT scale from 5.0 K to 24.5561 K. Reference SPRTs are calibrated on the ITS-90 using all fixed points within the sub-range of 13.8033 K to 273.16 K. Since 13.8033 K (e-H_2_ TP) and 24.5561 K (Ne TP) are calibration points for both the SPRT sub-range as well as for the ICVGT, the two reference scales agree at these points to within the stated uncertainty for the calibration. For temperatures above 13.8033 K, NIST disseminates the SPRT definition of the ITS-90 using the hydrogen vapor pressure definition for the points near 17.0 K and 20.3 K.

This same definition is also available as an SRM in the form of a NIST-calibrated capsule SPRT over the range 13.8 K to 430 K. The SRM 1750 [97] incorporates a calibrated capsule SPRT and an adapter probe for use in immersion-type fixed-point cells such as triple point of water cells. These SRMs are available to customers through the Standard Reference Materials Program for immediate use. This eliminates the need to wait for NIST cryogenic batch comparison calibrations to be scheduled.

The NIST calibration uncertainties for RIRTs, as well as for the lowest three SPRT sub-ranges, have been revised recently according to the most recent NIST ITS-90 realization results. These expanded uncertainties (*k* = 2) do not exceed 0.7 mK between 0.65 K and 273.16 K. A detailed assessment of the calibration uncertainties for capsule thermometers is presented in the NIST internal report NISTIR 6138 [98]. Table 3 gives the ranges of calibrations of CSPRTs, the expanded uncertainty *U* at the fixed points, and the maximum uncertainty over the various ranges from 13.8033 K to 505.078 K [99]. Information similar to that provided for CSPRTs is given for RIRTs and Germanium Resistance Thermometers (GRTs) for the ranges from 0.65 K to 84 K in Table 4 [99].

#### 6.2 Maintenance and Dissemination of the ITS-90 and Other Scales Above 83 K, Evaluations of Fixed-Point Cells, and Uncertainties of Calibrations Over the Range of Contact Thermometry

##### 6.2.1 Prior Scales

In the area of contact thermometry for this range of temperature, NBS/BS maintained the NHS by means of 16 special Hg-in-glass thermometers calibrated at 0 °C and 100 °C (centigrade) on the NHS. The ITS-27 and the ITS-48 were maintained by means of the oxygen boiling point, the ice point (0 °C), and the boiling points of H_2_O and S [100]. The IPTS-48(60) was maintained by the wire scale below 0 °C, by the triple point of H_2_O, the boiling point of H_2_O, and the freezing point of Zn. The triple point of H_2_O (0.01 °C) was introduced into the IPTS-48(60), replacing the ice point as a means of determining 0 °C. The IPTS-68 and the IPTS-68(75) were maintained by means of a wire scale below 0 °C, the triple point of H_2_O, and the freezing points of Sn and Zn [101].

Over this range of temperature, BS/NBS/NIST offered precise and accurate calibrations of thermometers. Between the times of the adoption of the IPTS-68 and the ITS-90, NBS/NIST also provided evaluation and certification of materials as SRMs and provided a measurement assurance program on the IPTS-68.

##### 6.2.2 The ITS-90

In this range of temperature, NIST maintains the ITS-90 through sets of fixed-point cells at each of the defining fixed points of the scale [25]. See Secs. 3.1.1.2 and 5.1 concerning the Ar TP apparatus.

NIST offers precise and accurate calibrations of thermometers, evaluation and certification of materials as SRMs, and provides a measurement assurance program. Also, customers’ fixed-point cells are evaluated.

###### 6.2.2.1 Calibrations

At NIST, long-stem and capsule SPRTs have been calibrated on the ITS-90 in the range 83.8058 K to 1234.93 K since the adoption of the scale in 1990 [25]. Over this range of temperature, NIST has the capability for precise and accurate calibrations of essentially any type of thermometer used in contact measurements. These include resistance thermometers of the usual types (standard and industrial grade) over their customary temperature ranges, both noble-metal and base-metal thermocouples, liquid-in-glass thermometers, and the various types of digital thermometers. The thermometers listed here are calibrated either directly against the ITS-90 defining fixed points or by comparison with thermometers that have been calibrated against the ITS-90 fixed points, whichever is appropriate.

The methods of calibration, temperature ranges of calibration, and the associated uncertainties for some of these thermometers are as follows.

####### 6.2.2.1.1 Resistance Thermometers

######## Standard platinum resistance thermometers

Since the scale came into effect in 1990, long-stem and capsule SPRTs have been calibrated on the ITS-90 in the range 83.8058 K to 1234.93 K, as appropriate, taking into account the effects of hydrostatic head and self-heating and, for FPs and MPs, any deviation of the gas pressure from 101 325 Pa. Table 2, given in Sec. 3.1.1.2, shows that the uncertainty of calibration at the fixed points, using current equipment and measurement practices, is highly satisfactory. Table 5 gives the ranges of calibrations of (HT)SPRTs, the uncertainties at the fixed points, and the maximum uncertainty over the various ranges up to 1234.93 K (961.78 °C) [99]. Table 3 gives similar information over the various ranges from 83.8058 K to 505.078 K for CSPRTs [99].

######## Industrial platinum resistance thermometers (IPRTs) and thermistors

NIST calibrates IPRTs within the range from 77 K to 835 K (−196 C to 552 °C), as desired by the customer, and thermistors over any part of the range from 77 K to 435 K (−196 °C to 162 °C). Since the resistance-temperature relationships of thermistors are essentially exponential, the total range for any given thermistor is not large. The various ranges available and the uncertainties for those calibrations are given in Table 6 [102].

####### 6.2.2.1.2 Thermocouples

The types of thermocouples, the methods of calibration, the ranges of calibration and the uncertainties of calibration that are offered at NIST are given in Table 7 [103]. Note also that NIST has the capability to calibrate types of thermocouples other than those indicated in Table 7, e.g., Au/Pt and W/Re thermocouples.

####### 6.2.2.1.3 Liquid-in-Glass Thermometers

NIST has the capability of calibrating both total immersion and partial immersion liquid-in-glass thermometers over their entire range. The information concerning these calibrations is given in Table 8 [104]. Note that NIST does not calibrate household-type thermometers, nor does it calibrate fever thermometers; it calibrates only precision-type scientific thermometers.

####### 6.2.2.1.4 Digital Thermometers

NIST has the capability of calibrating digital thermometers (quartz, resistance and thermocouple types) over their entire range. The information concerning those calibrations is the same as that given in Table 6 [102], except the uncertainty may be limited by the digital display.

###### 6.2.2.2 Non-Uniqueness

The uncertainty of calibration is sufficiently small for the (HT)SPRTs, CSPRTs and RIRTs to determine the differences in indicated temperatures as given by the different thermometers at temperatures between the fixed points, i.e., the non-uniqueness of the ITS-90 [13]. The estimated uncertainty of the ITS-90 due to non-uniqueness is given to be within ± 0.5 mK between 13.8 K and 273 K; within ± 1 mK from 273 K to 693 K; within ± 3 mK from 693 K to 933 K; and within ± 5 mK from 933 K to 1235 K [20]. Experiments are in progress at NIST to determine the non-uniqueness of HTSPRTs between 900 K and 1235 K. *As a cautionary note*, we point out that due to flaws in the capillary, there could be a small non-uniqueness between calibration points in liquid-in-glass thermometers also.

###### 6.2.2.3 Evaluation of Customer Fixed-Point Cells

In addition to calibration of thermometers, we evaluate fixed-point cells of Ar (83.8058 K), Hg (234.3156 K), water (273.16 K), Ga (302.9146 K), In (429.7485 K), Sn (505.078 K), Zn (692.677 K), Al (933.473 K) and Ag (1234.93 K). The expanded uncertainty (*k* = 2) of these evaluations are at the 0.1 mK to 1 mK level.

###### 6.2.2.4 Measurement Assurance Program (MAP)

We also offer a service that evaluates the complete measurement system of the participant. This is done through a Measurement Assurance Program (MAP) in which we send to the participant a set of three calibrated thermometers, which the participant then calibrates according to his/her own procedures. The participant returns the thermometers to NIST along with his/her own results. After a re-calibration of the thermometers, the participant’s results are analyzed in comparison with the NIST results. The results include details about systematic errors in the participant’s laboratory, as well as statements on the participant’s other uncertainties. The participant is sent the results of the analysis.

###### 6.2.2.5 Standard Reference Materials (SRMs)

As part of a program to disseminate the ITS-90, NIST provides not only a calibration service but also thermometric fixed-point cells, high-purity metals for constructing fixed-point cells, and thermometers that are certified as SRMs for the temperature range from −259.3476 °C to 1768 °C. SRM high-purity metals of mercury, gallium, indium, tin, zinc, aluminum and silver, with mass fractions ≥ 99.9999 %, may be used to construct ITS-90 fixed-point cells that cover the temperature range from −38.8344 °C to 961.78 °C. SRMs of large freezing-point cells of tin (231.928 °C) and zinc (419.527 °C) are for use in calibrating SPRTs in accordance with the ITS-90. SRMs of small fixed-point cells are for use in calibrating small thermometers, e.g., thermistors and IPRTs, over the temperature range from 29.7646 °C to 156.5985 °C. SRM thermometers (capsule SPRT, clinical laboratory, Pt thermoelement and Au/Pt thermocouple) have been calibrated in terms of the ITS-90 and cover the temperature range from −259.3476 °C to 1768 °C.

####### 6.2.2.5.1 SRMs of ITS-90 Fixed-Point Metals

A single lot (20 kg to 89 kg) of high-purity metal (mass fraction ≥ 99.9999 %) for each of the seven metals that define the ITS-90 from −38.8344 °C to 961.78 °C has been certified as fixed-point-standard SRMs. Table 9 shows the SRM number assigned to each metal, the freezing-point temperature of each metal, the SRM unit sample size, the purity of the sample, and the expanded uncertainty (*k* = 2) associated with the freezing-point temperature of the SRM metal in fixed-point cells (as tested using randomly-selected samples from the lot). These SRMs were developed for the fabrication of freezing-point cells of the ITS-90 defining fixed points and for their use in the calibration of (HT)SPRTs and other thermometers requiring high-accuracy calibrations. In the case of SRM 740 and 741, these SRMs were replaced with higher-purity, tear-drop, shot samples designated SRM 740a and 741a, respectively. Further information can be found in Refs. [26,105–107].

####### 6.2.2.5.2 SRMs of Large ITS-90 Fixed-Point Cells

Large SRM fixed-point cells containing high-purity (mass fraction ≥ 99.9999 %) Sn and Zn were developed for use as ITS-90 defining fixed-point cells to calibrate (HT)SPRTs. The cells were fabricated and certified by measurements in the manner described in Ref. [27]. They are designated as SRM 1747 (Sn freezing-point cell) and SRM 1748 (Zn freezing-point cell). They, together with the triple point of water cell, are used to calibrate SPRTs from 0 °C to 420 °C, used for part of the calibration of SPRTs from 0 °C to 661 °C, or used for part of the calibration of HTSPRTs from 0 °C to 962 °C.

Table 10 shows the serial number (s/n) assigned to each SRM freezing-point cell and the expanded uncertainties (*k* = 2) assigned to the freezing-point temperature of the cells. Figure 9 shows a cutaway drawing of the fixed-point cells. The distance from the inside bottom of the graphite re-entrant well to the top of the liquid level of the metal of the cells is 20.5 cm. These fixed-point cells are designed to fit into most commercially-available fixed-point-cell furnaces.

####### 6.2.2.5.3 Small SRM Fixed-Point Cells

A series of six small, sealed fixed-point cells were developed to cover the temperature range from 29.7646 °C to 156.5985 °C for the purpose of calibrating small thermometers and for use as check points in which to verify the calibration of small thermometers (e.g. thermistors, diode thermometers, industrial-grade resistance thermometers). Materials were chosen that have a freezing-point, melting-point, or a triple-point temperature, closely matching critical temperature values used in clinical, biomedical, or pharmaceutical laboratories as reference-temperature check points. Additionally, these cells may be used for the calibration of thermometers that do not adhere to the definition of the ITS-90. Two of these SRM cells (SRM 1968 and SRM 1971) contain high-purity metals that have defining fixed points on the ITS-90 and the other four are secondary fixed points. Table 11 gives the SRM number, sample material, cell type, fixed-point temperature, and expanded uncertainty (*k* = 2) of the fixed-point temperature of the cell.

####### 6.2.2.5.4 SRM Thermometers

Four different SRM thermometers were developed as a means to disseminate NIST-calibrated devices to cover the temperature range from −259.3476 °C to 1000 °C. Each of these four SRMs was chosen for specific areas of industrial interest. Table 12 gives the SRM device number, thermometer type, usable temperature range and uncertainty in the measured temperature associated with the SRM.

### 7. Future Work in Contact Thermometry

Work is in progress and/or planned in areas described below.

There is a large discrepancy in the *PV* gas thermometry measurements in the range 500 K to 800 K on which the ITS-90 is based. The acoustic thermometry work will be continued to determine the difference (*T* − *T*_90_) from 273 K to 800 K to resolve the discrepancy. See Sec. 4.1.

Work will continue to develop JNT utilizing the latest advances in digital noise processing techniques, in conjunction with the advances in the Josephson pulse-quantized voltage sources, to achieve relative uncertainties of 1 × 10^−5^ between 83.8 K and 430 K. See Sec. 4.2.

Thermocouple thermometry is relatively simple to perform. Significant improvement in accuracy has been realized recently with pure metal thermocouples, i.e., Au/Pt and Pt/Pd thermocouples, which do not suffer alloy composition variation along the wire or preferential oxidation or vaporization. The use of Au/Pt and Pt/Pd thermocouples has been accepted but procedures for optimal application of them still must be developed. See Sec. 5.3.

An area of work in progress is the development and application of thin-film (< 1 μm) thermocouples which have fast response and do not perturb the object being measured. A number of thermocouple materials, including intermetallics, have been investigated. The application of the thin-film thermocouples for calibrating other thermometers, e.g., light-pipe radiation thermometers, is an on-going activity. See Sec. 5.3.

Work is in progress on developing various cryogenicgas fixed points (triple points) in sealed cells for testing the non-uniqueness of the ITS-90 and for the international comparison of the temperature scale through exchange of sealed cells. One of the active areas is the international comparison of the triple point of D_2_, with the D_2_ sample as free of HD and H_2_ as possible. See Sec. 5.1.

The ITS-90 has been realized. The capability below 83.8 K is being closely monitored to improve the realization and to be able to calibrate NIST standard reference thermometers for calibrating other thermometers when the necessity arises.

As part of an effort to determine the non-uniqueness of the ITS-90, HTSPRTs are being compared in the range 600 °C to 970 °C and will be continued in lower ranges. See Sec. 6.2.2.2.

The work to improve and maintain the calibration of (HT)SPRTs, directly against fixed points in the range 83 K to 1235 K, and of CSPRTs and RIRTs, by comparison techniques in the range 0.65 to 83.4 K, for the nation’s thermometry community is an on-going activity. This work also supports the active liquid-in-glass, IPRT, thermistor, digital thermometer, and low-temperature thermocouple calibration programs by providing calibrated SPRTs for use as the reference thermometer. The calibration of thermocouples from 0 °C to the Au FP is continuing.

Semi-annual precision thermometry seminars are also an on-going effort of the NIST Thermometry Group.

## Part II. Non-Contact (Radiation) Thermometry

### 8. Introduction

This part discusses the non-contact method of determining temperature from measurements of the radiant flux; this technique is often termed radiation thermometry. The quantity realized can be either the thermodynamic temperature or a value on the ITS-90, depending on the measurement techniques (see Sec. 1 in Part I for more on thermodynamic temperature). To realize thermodynamic temperature from measurements of radiant flux, the radiation thermometer must be a primary device, that is, the equation of state does not depend on unknown, temperature-dependent parameters. Examples of both of these methods are given in Secs. 9 and 10.

There are several quantities in radiometry that will be mentioned below, so here we provide a brief introduction; a summary is given in Table 13. Radiant flux or power is sensed by a detector that operates as a transducer using various physical mechanisms. The geometric extent of the radiant flux is defined by some optical system that includes the source and the detector and often includes defining apertures and other optical elements. Irradiance is the radiant flux incident on a surface divided by the area of that surface (i.e., radiant flux per area), where the surface can be real (e.g. the aperture in front of the detector), or arbitrary (e.g., as a function of distance from a point source). Radiance is the radiant flux in a beam per area and solid angle of that beam. It is often the case that the area in question refers to the source of radiant flux. Exitance is the radiant flux emitted into the entire hemisphere above the radiating surface. All of these quantities exhibit spectral dependence, so an additional qualifier is necessary: “spectral” refers to the amount of radiation per wavelength.

We begin with a brief historical review, then discuss the calibration services and current related research in the Optical Technology Division at NIST, and conclude with a look to the future.

### 9. Historical Developments

Accurate determinations of material temperatures using non-contact, optical methods have long played critical roles in history. For example, some of the very early practitioners of optical pyrometry were the earliest metal workers, who visually determined the correct temperature at which to begin forming or tempering their metal implements. In general, the radiative method of temperature determination is utilized when the temperatures are too high for the materials used with other types of thermometers or the object is remote or otherwise inaccessible (in motion, for example). The relationship between the temperature and the radiative properties of the material were not fully understood until Planck published his critical papers in 1900 and 1901 [120]. The importance of Planck’s contributions is well known. The formation of NIST in 1901 (as the National Bureau of Standards) coincided with the beginnings of quantum physics that were initiated by Planck’s discoveries.

Before discussion of the historical development of non-contact thermometry at NIST, the relationship between radiance temperature and temperature, which de pends on the spectral emissivity of the source, *ε*(*λ*), must be explained. Spectrally-selective pyrometers or optical radiometers measure some spectral portion of the radiance called spectral radiance *L*(*λ*) which is described by Planck’s law,

$$L\left(\lambda \right)=\frac{c_{1L}}{n^{2}\lambda^{5}}\frac{\varepsilon \left(\lambda \right)}{\exp\left[c_{2}/\left(n\lambda T\right)\right]-1},$$ (5)

where *c*_1L_ is the first radiation constant for spectral radiance, *c*_2_ is the second radiation constant, *n*(λ) is the index of refraction of air, and *T* the thermodynamic temperature of the source. If the spectral emissivity of the source, *ε*(λ), is set to one, then the temperature derived from Eq. (5) becomes the radiance temperature, *T*_R_, of the source. Thus, it is critical to have knowledge of the spectral emissivity of the material before the thermodynamic temperature can be inferred from the measurement of radiance temperatures.

The radiant power per area emitted by a source at all wavelengths into the hemisphere is the total exitance *M*. For an ideal blackbody (*ε* = 1) at temperature *T*, the exitance is derived from integration of Eq. (5):

$$M=n^{2}\sigma T^{4},$$ (6)

where *σ* is the Stefan-Boltzmann constant, and Eq. (6) is known as the Stefan-Boltzmann law. Spectrally-flat radiometers, with uniform response over a broad range of wavelengths, are used to measure *M*. In Eq. (6), the spectral dependence of the index of refraction was neglected, *n*(*λ*) ≈ *n*; for air, it is often sufficient to approximate further, with *n*^2^ ≈ 1. If the emissivity is not unity, but is independent of wavelength, then the exitance of this graybody follows Eq. (6) but is reduced in proportion to the emissivity *ε*. The values for *c*_1L_, *c*_2_, and *σ* are given in Table 14.

In either the spectral or total mode of measurement, the ideal blackbody functions as a primary standard because the equations of state are known in terms of the underlying physics, and there are no parameters that depend on temperature. Practically speaking, the inaccuracy of the measurements is determined by the degree to which the blackbody source and the radiometer perform in ways that can be modeled. Examples of inevitable effects include temperature gradients in the blackbody, instability of the reflectance of the cavity walls, diffraction and scatter of the radiant flux, and the spectral, temporal, spatial response functions of the radiometer.

W. W. Coblentz [121] and G. K. Burgess [122] performed the earliest work at NIST on blackbody radiation and the accurate measurement of radiance temperature. Coblentz was especially prolific, working to measure the Stefan-Boltzmann constant [123] using an early precursor for absolute detectors [121]. Later, this work was directed to spectroradiometry, with accurate determinations of radiance temperatures required for calibration of carbon-filament lamps [124]. The transfer standards were used, just as at present, to calibrate spectroradiometers to measure spectral irradiance [125]. Years later, D. C. Ginnings and M. L. Reilly developed a NIST cryogenic electrical substitution radiometer with the goal of determining the freezing-point temperature of gold from the ratio of the exitance to that from a black-body at the triple point of water [126]. Characterization of the system revealed unacceptably large systematic effects from diffraction and scatter but no steps were taken at that time to rebuild the apparatus (but see below).

From Eq. (6), it is clear that absolute determination of the exitance from a blackbody at a known temperature provides a measure of the Stefan-Boltzmann constant. T. J. Quinn and J. E. Martin, at the National Physical Laboratory in the United Kingdom, used a blackbody at the triple point of water and a cryogenic electrical substitution radiometer to determine *σ* with a relative standard uncertainty of 0.0134 % [127]. This radiometrically-measured value agreed to within the combined uncertainties with the value derived from fundamental constants (Ref. [128] and Table 14), but the uncertainty in this measured value is about 19 times larger than that derived from the fundamental constants. A significant outcome of performing these and other difficult temperature experiments (see Ref. [129] and references therein) was refinements in the field of cryogenic electrical substitution radiometry. Often termed absolute cryogenic radiometers (ACRs), these devices now function as the primary standard, by way of stabilized laser radiation, for spectral flux calibration of detector standards (see for example Ref. [130]).

As mentioned previously, human vision served as the first radiometer for radiometric temperature determinations. At the turn of the 20th century, it became possible to quantify the results with the invention of the disappearing-filament pyrometer [131]. This device has an incandescent filament that is viewed through a red filter in conjunction with the material under test. The pyrometers are calibrated using a blackbody by adjusting the electrical current in the filament until the human observer cannot distinguish it from the blackbody radiation. The resulting current-to-temperature relationship is critically dependent on the training and experience of the operator. Disappearing-filament pyrometers are still in use today; they are calibrated at NIST from 800 °C to 4200 °C.

In the middle of the 20th century, researchers began to utilize photomultiplier tubes (PMTs) in optical pyrometers. These photoelectric pyrometers, which generally include spectrally-selective components to limit the wavelength range to a few nanometers, are calibrated using a fixed-point blackbody at a known temperature (see below). The temperature of the test blackbody is a function of the ratio of the spectral radiance of the test to the fixed-point blackbody. Above the freezing point of silver, 961.78 °C, the ITS-90 defines temperature in terms of these spectral radiance ratios, where the reference blackbody can be that at the freezing-point temperature of silver, gold, or copper [1]. The first NIST photoelectric pyrometer, described by Lee [132], has features common with the device used today [133].

**Fig. 17:**
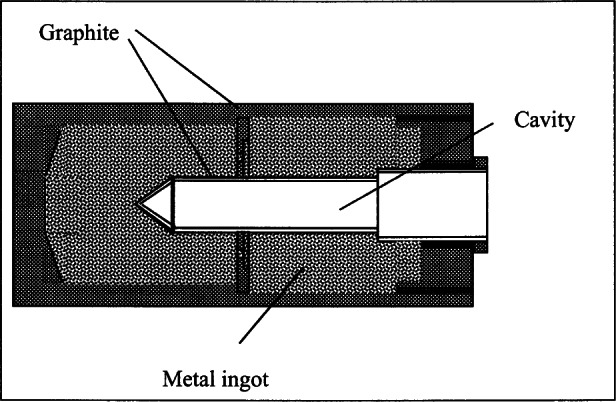
Schematic of a crucible of a freezing-point blackbody.

A fixed-point blackbody source operates at the unique temperature of the equilibrium liquid to solid phase transition in pure metals. The blackbody cavity is surrounded by the metal ingot, which in turn is contained in a mechanical structure (the crucible) that is sealed to the front portion of the blackbody cavity. The assembled crucible, shown in Fig. 17, is operated in a resistively-heated furnace. For indium, tin, aluminum, silver, gold, or copper reference blackbodies, the cavity and the crucible are machined from high-purity, high-density graphite. Lee has described the early NIST implementation [134]; the current implementation includes an alkali-metal heat-pipe furnace liner to limit the temperature gradients along the crucible [135]. The metal is kept pure during assembly using a vacuum processing technique [136].

Because the fixed-point blackbody sources are tedious to operate and are not easily portable, radiance temperature lamps are used as secondary standards. The vacuum tungsten-strip lamp of the Quinn-Lee design [137] was developed at NIST and used in the international temperature comparison of the IPTS-68 [138]. The vacuum strip lamps were stable, drifting less than 0.1 K per 1000 h of operation, but they cannot be operated at high currents or the tungsten will evaporate. At 655 nm the maximum radiance temperature is about 1700 °C. Gas-filled tungsten-strip lamps can be operated at higher currents than the vacuum strip lamps; at 655 nm, the maximum radiance temperature is about 2300 °C.

### 10. Current Work at NIST in Non-Contact Thermometry

#### 10.1 Calibration Capabilities

The effort in radiance temperature at NIST can be divided into routine calibrations and radiance temperature research. Both detectors and sources are calibrated over the temperature range from 60 K to 3000 K. Three main facilities exist for calibrations: The Low-Background InfraRed (LBIR) facility, the Low-Level Temperature (LLT) facility, and the NIST Radiation Temperature Calibration Laboratory (RTCL). The LBIR calibration facility is capable of measuring blackbody temperatures from 60 K to about 1000 K in a low background 20 K environment [139]. The LLT facility, with variable-temperature water- and oil-bath blackbodies and cesium and sodium heat-pipe blackbodies, has been developed for calibration of pyrometers and sources in the temperature range from 288 K to 1223 K [140–142]. The LLT facility also has various fixed-point black-bodies ranging from gallium (302.9146 K) to gold (1337.33 K). The third facility is the NIST RTCL for the dissemination of the ITS-90 [133]. Each of these facilities establishes traceability to SI quantities in different ways. The LBIR facility relates the quantity of radiant flux to electrical standards; the LLT and RTCL facilities relate the quantity of radiant flux to the ITS-90.

In the LBIR facility, the blackbody radiance temperatures are determined using an ACR at 2 K with a precision aperture set at a known distance from a blackbody that has a second precision aperture. The measurements are done in a vacuum chamber with a pressure of 1.3 × 10^−6^ Pa or less when the chamber is at 270 K. During operation, cryoshrouds that form the interior walls are cooled to 20 K using He gas. The radiance temperature is found from Eq. (6), the Stefan-Boltzmann law, and a geometric factor that accounts for the less-than-hemispherical collection geometry:

$$T_{R}={\left(\frac{\phi }{\sigma F_{1}\pi {r_{1}}^{2}}\right)}^{1/4},$$ (7)

where *ϕ* is the optical power (radiant flux), $F_{1}=\frac{1}{2}\left(z-{\left(z^{2}-4x^{2}y^{2}\right)}^{1/2}\right)$ is the configuration factor with $x=\frac{r_{2}}{s}$, $y=\frac{s}{r_{1}}$, and *z* = 1 + (1 + *x*^2^)*y*^2^, *s* is the distance between the apertures, and *r*_1_ and *r*_2_ are the radii of the blackbody and the ACR apertures, respectively. Since the blackbodies are characterized in the range of temperatures and wavelengths where diffraction effects become significant, the optical power in Eq. (7) is corrected for diffraction. Accurate diffraction corrections were determined at NIST by E. L. Shirley [143]; the computer code is available on diskette for the general use of the radiometry community [144]. Because the uncertainties in the diffraction corrections and the uncertainties caused by geometric alignment depend on the aperture sizes, the final radiance temperature uncertainty assignments depend on aperture size (Table 15). For the smallest apertures at the lowest temperatures, the large uncertainties in the measured signals preclude measurements of radiance temperatures.

In the LLT facility, the temperatures of the water- and the oil-bath blackbodies are determined using a platinum resistance thermometer or a thermistor that is immersed in the stirred bath [140, 141]. The temperatures of the cesium and the sodium heat-pipe blackbodies are determined using Au/Pt thermocouples that are inserted into wells along the outside of the cavities [142]. The emissivity of the blackbodies under isothermal conditions is calculated using a Monte Carlo analysis [145] and verified experimentally by measurement with different source apertures. Preliminary comparisons between the Ag and the Al fixed-point blackbodies and the cesium heat-pipe blackbody agree to within 60 mK [146]. The capabilities of the LLT facility are summarized in Table 16 and the uncertainty budget for the pressure-controlled heatpipe-blackbody source is given in Table 17.

**Fig. 18:**
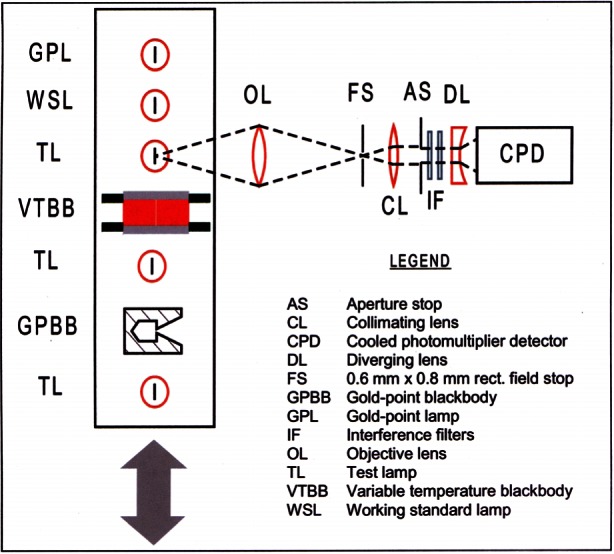
Schematic of the NIST Radiation Temperature Calibration Laboratory, with the various sources mounted on a translation table. The PEP consists of the objective lens, field stop, collimating lens, aperture stop, interference filter, diverging lens, and cooled photomultiplier detector.

In the NIST RTCL, illustrated in Fig. 18, the radiance temperature scale above 800 °C is disseminated using the NIST photoelectric pyrometer (PEP) with a gold-point blackbody, a vacuum strip lamp of the Quinn-Lee design, and a variable temperature blackbody (VTBB). The temperature of the Quinn-Lee strip lamp is determined by spectral radiance ratios to the gold-point blackbody, and the temperature of the VTBB is determined by ratios of its spectral radiance to that of the Quinn-Lee lamp. The spectral response of the PEP is centered at 655.3 nm with a full-width-half-maximum of 1.1 nm using two interference filters. The field stop in the PEP limits the target area to a rectangle 0.6 mm by 0.8 mm.

The simplified measurement equation for the calibration of the Quinn-Lee lamp, which has a radiance temperature of about 1530 K, is

$$\frac{L_{\lambda }\left(T_{R}\right)}{L_{\lambda }\left(T_{\text{Au}}\right)}=\frac{1}{\varepsilon_{\text{Au}}}\frac{\exp\left(c_{2}/\left(n\lambda T_{\text{Au}}\right)\right)-1}{\exp\left(c_{2}/\left(n\lambda T_{R}\right)\right)-1},$$ (8)

where *T*_Au_ is the temperature of the gold freezing-point, *T*_R_ is the radiance temperature of the Quinn-Lee lamp, and λ is the mean effective wavelength of the PEP. Evaluation of the index of refraction *n* as a function of wavelength is given as Eq. (7) in Ref. [133]; that reference also gives *ε*_Au_ = 0.9999. A similar, but not identical, version of Eq. (8) applies for the calibration of the VTBB using the Quinn-Lee lamp.

For temperatures above the freezing point of silver at 961.78 °C, the NIST procedure is an implementation of ITS-90, with a subtle difference regarding the determination of the value for the temperature at which gold freezes. The ITS-90 value for the freezing point of gold (or silver or copper) was achieved by a critical compilation of experimental values, but no uncertainties were included. It is possible, by direct measurement of the radiant flux, to determine the freezing-point temperature radiometrically, without reference to gas thermometry. Using a room-temperature electrical substitution radiometer and a calibrated p-n silicon photodiode, the freezing temperature of gold was determined to be 1337.33 K with an expanded uncertainty (*k* = 2) of 0.23 K [135]. This value is in agreement with the ITS-90 assignment, and the uncertainty is included in the uncertainty budget for the NIST radiance temperature and spectroradiometric scales. The result is termed the 1990 NIST Radiance Temperature Scale [147].

The standard measurements in the RTCL are calibrations of tungsten-ribbon filament lamps, disappearing-filament optical pyrometers, and portable radiation thermometers. Other tests can be done as a special request depending on the capabilities of the RTCL. Tungsten-ribbon filament lamps are calibrated at 655.3 nm by comparison to the Quinn-Lee lamp. The lamps to be measured are either vacuum tungsten-strip lamps, which can be calibrated from 800 °C to 1700 °C, or argon-filled lamps, which can be calibrated from 800 °C to 2300 °C. Typical uncertainties for the latter are given in Table 18 [133]. The disappearing-filament optical pyrometers and the portable radiation thermometers are calibrated using the VTBB, which has an experimentally-determined emissivity of 0.9995. Typical uncertainties for a radiation thermometer are given in Table 19.

#### 10.2 Research in the Field of Radiance Temperature

NIST uses a variable temperature blackbody as a standard of spectral radiance to provide its spectral radiance and irradiance calibration services [148, 149]; the black-body temperature is also determined using a gold-point blackbody and spectral radiance ratios. Therefore, the uncertainty in the temperature determinations affects the uncertainty of the spectroradiometric quantities, and a critical objective at NIST is to reduce both of these uncertainties. The standard lamps of spectral irradiance, 1000 W FEL lamps^2^, have spectral shapes similar to a 3000 K blackbody source in the ultraviolet and visible wavelength regions. Due to the spectral mismatch, five intermediate steps are necessary to calibrate the spectral irradiance standards with concomitant increase in the total uncertainties at each step. A method to reduce the uncertainties of the spectral irradiance is to directly determine the radiance temperature of a large-area, high-temperature blackbody (HTBB) at 3000 K and then use the output of the blackbody to calibrate the 1000 W FEL lamps directly.

The radiance temperature of a HTBB can be determined in two different ways. The first, called the ITS-90 method, is to follow ITS-90 and use spectral radiance ratios of the HTBB and the gold-point blackbody (or silver or copper). The radiance temperature uncertainty *u* for the HTBB is related to the radiance temperature uncertainty of the fixed-point blackbody by

$$u\left(T_{\text{BB}}\right)=\frac{u\left(T_{\text{FP}}\right)}{T_{\text{FP}}^{2}}T_{\text{BB}}^{2},$$ (9)

where *T*_FP_ is the temperature of the fixed-point black-body and *T*_BB_ is the temperature of the HTBB and their standard uncertainties are *u*(*T*_FP_) and *u*(*T*_BB_) respectively. Therefore the limiting uncertainty for the ITS-90 method is *u*(*T*_BB_) ≈ 5*u*(*T*_FP_) for *T*_BB_ = 3000 K.

A second, more direct approach, is to determine the radiance temperatures of the blackbody with transfer radiometers that are calibrated using ACRs. In the LBIR calibration facility, this approach is direct, in that there are no transfer radiometers and both the source and detector are in the same vacuum chamber. For the HTBBs in the LLT and RTCL facilities, transfer radiometers that function as secondary standards are used. In principle, the HTBB could be calibrated with an independent (and dedicated) ACR, but NIST has adopted the approach of using the High Accuracy Cryogenic Radiometer (HACR) [150] as a central device and silicon photodiode trap detectors or other detectors to disseminate the spectral flux responsivity scale. The candela (SI base unit for luminous intensity) and detector spectral flux responsivity are realized this way [130], and it is the goal of the radiation temperature research to do the same for the radiance temperature, spectral radiance, and spectral irradiance scales [151–153].

**Fig. 19:**
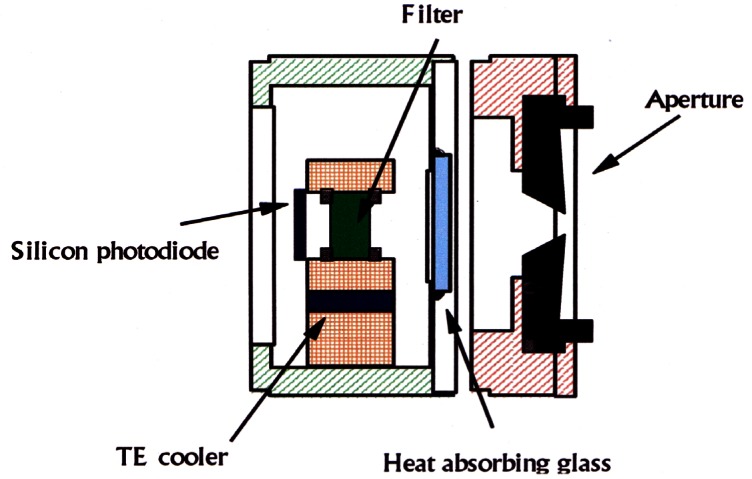
A schematic of the filter radiometers for measurements of spectral irradiance. The main components are the precision aperture, which is about 4 mm in diameter, various filters to limit the spectral bandpass, and a silicon photodiode. The bandpass filters are either interference or absorbing glass design. The electronics are not shown.

One type of transfer radiometer is a filter radiometer for spectral irradiance that is calibrated against the HACR; a schematic is given in Fig. 19. If the filter radiometer is calibrated for spectral power responsivity, *R*_E_(λ), in units of A/W, then the radiance temperature and measured signal are related through the measurement equation

$$\begin{array}{r} r_{E}=G\pi r_{2}^{2}\int R_{E}\left(\lambda \right)E\left(\lambda \right)d\lambda \\ =G\pi r_{1}^{2}F_{1}\int R_{E}\left(\lambda \right)\pi L\left(\lambda ,T_{R}\right)d\lambda , \end{array}$$ (10)

where *E*(λ) is the spectral irradiance at the radiometer aperture, *G* is the gain in V/A, and *r*_E_ is the signal from the radiometer in volts. Since all the geometric factors in *F*_1_ can be measured, Eq. (10) can be solved iteratively for the radiance temperature that produces the measured signal.

**Fig. 20:**
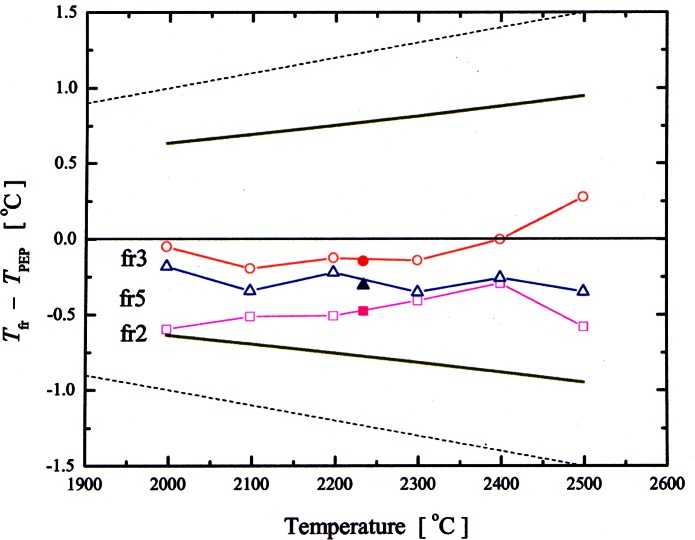
The differences in the temperature of a high-temperature blackbody determined using ITS-90 (labeled *T*_PEP_) and three irradiance filter radiometers (labeled *T*_fr_). The centroid wavelengths of the filter radiometers are 405 nm, 469 nm, and 560 nm, with effective bandwidths of 57 nm, 114 nm, and 107 nm, respectively. The solid lines represent the expanded (*k* = 2) component of uncertainty from the uncertainty in the freezing point of gold; the dashed lines represent the total expanded uncertainty (*k* = 2) in the ITS-90 realization.

The agreement between the radiance temperatures of a HTBB determined using the ITS-90 method and the absolute radiometric method has recently been established. The radiance temperature of the VTBB in the RTCL was determined with the PEP using radiance ratios against the gold fixed-point blackbody. At the same time, six filter radiometers, with center wavelengths between 350 nm and 950 nm, measured the flux from the VTBB. The agreement between the two determinations in the temperature range between 2200 K and 2800 K was found to be closer than 0.5 K [151]; see Fig. 20.

**Fig. 21:**
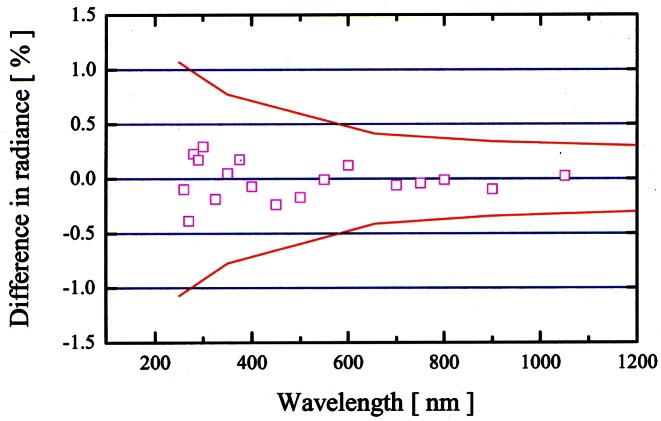
The percent difference, as a function of wavelength, in spectral radiance of a high-temperature blackbody determined using a tungsten-strip lamp and the irradiance filter radiometers. The solid lines represent the expanded uncertainty in the NIST spectral radiance scale.

The level of agreement between the current NIST spectral radiance scale and the spectral radiance assigned to a large area HTBB was also established using filter radiometers [152]. The radiance temperature of the HTBB was determined using filter radiometers by direct measurement of the radiant flux. At the same time, the radiance temperature was determined by spectral radiance ratios using an argon-filled tungsten-strip lamp. The lamp was calibrated for spectral radiance in the NIST Facility for Spectroradiometric Calibrations (FASCAL) using the method of ITS-90 and a gold freezing-point blackbody. The two independent methods of determining spectral radiances were found to agree to within 0.5 % from 250 nm to 1050 nm, which is within the combined uncertainties of the comparison [152]. These results are illustrated in Fig. 21.

### 11. Future Directions in Non-Contact Thermometry

To date, filter radiometers such as the ones described above could be calibrated for spectral flux responsivity only by using the NIST Spectral Comparator Facility [154]. With this facility, absolute spectral flux responsivity determinations require that the entrance pupil of the filter radiometer be underfilled. For the spectral irradiance responsivity, determination of the area of the aperture is necessary from ancillary measurements [155]. The Spectral Comparator Facility uses monochromators to spectrally select the output of continuum sources, and the response of the test detector is determined by comparison to a standard transfer detector [154]. The instrumental bandwidth of the monochromator can be varied, but settings below 1 nm generally result in inadequate signal-to-noise ratios. The wavelength uncertainty is about 0.1 nm. The combined uncertainties of *R*_E_(λ) depend on wavelength, and increase rapidly below 450 nm and above 900 nm [154].

**Fig. 22:**
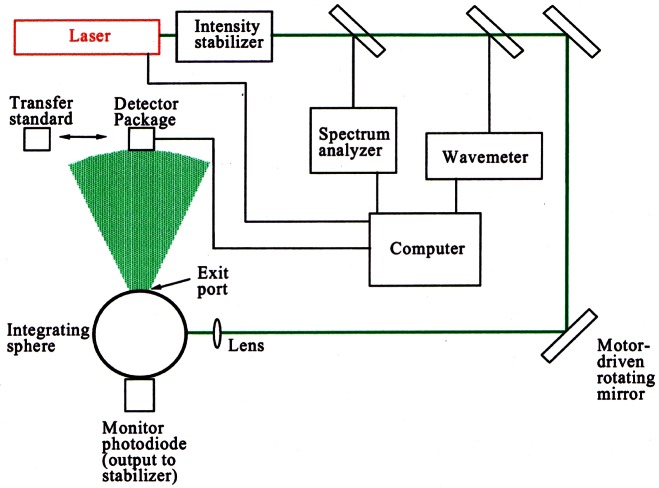
A schematic of SIRCUS, illustrating the flux stabilized laser sources that are input to an integrating sphere to create a uniform, monochromatic source of spectral radiance. The spectral radiance is determined from the transfer standard, the area of its precision aperture, the area of the precision aperture at the exit port of the sphere, and the distance between the two apertures. The radiometer, labeled “detector package,” can be calibrated either for radiance or irradiance measurements.

In an effort to reduce the uncertainties in absolute spectral irradiance response and to provide a new facility for spectral radiance response, the Optical Technology Division at NIST has developed a Spectral Irradiance and Radiance Responsivity Calibrations with a Uniform Source (SIRCUS) facility that is based on tunable lasers [156–158]. The lasers are coupled to integrating spheres that have high throughput. The laser-illuminated sphere source provides a high flux, lambertian, tunable, monochromatic light source, and the test detector is calibrated by direct comparison to a standard irradiance detector. The experimental components are illustrated in Fig. 22. The response of this detector is known from measurements of its aperture area [155] and spectral flux calibration with the HACR. SIRCUS results to date compare favorably with the previous method, and the uncertainty was improved by a factor of two or more for a narrowband radiance filter radiometer [159]. Results from the SIRCUS calibrations of the irradiance-measuring filter radiometers used for the spectral irradiance scale realization are under investigation, and it is anticipated that the uncertainties in the NIST spectral irradiance scale will be reduced by up to a factor of five, depending on wavelength.

SIRCUS will result in a new generation of pyrometers that are calibrated without dependence on black-body radiation. Such absolute pyrometers would determine the radiance temperature of blackbodies using

$$r_{L}=G\int R_{L}\left(\lambda \right)L\left(\lambda ,T_{R}\right)d\lambda ,$$ (11)

where *R*_L_(λ) is the absolute radiance response and *r*_L_ is the signal from the radiometer. The radiance temperature of the blackbody is solved iteratively using Eq. (11) and the measurements for *R*_L_(λ) and *r*_L_. The uncertainties in radiance temperature that result from the uncertainty in the spectral radiances follow from the derivative of Planck’s law in the Wien approximation,

$$\frac{dL}{L}=\frac{c_{2}}{\lambda }\frac{dT_{R}}{T_{R}^{2}}.$$ (12)

Future measurements with SIRCUS should result in a relative uncertainty in the spectral radiance response of about 0.05 % (*k* = 2) from 400 nm to 2000 nm. If this is achieved, the uncertainty component in radiance temperature arising from the uncertainty in radiance would be 23 mK (*k* = 2) at 1000 K and 650 nm (Table 20). At the gold point, this uncertainty component would be about 40 mK at 650 nm, which is about five times smaller than the combined expanded uncertainty of the previous NIST measurement [135]. Of course, the contributions from the other uncertainty components for these new pyrometers (such as measurement repeatability, non-linearity, size-of-source effect, and temporal stability) must be < 20 mK for the results to be an improvement over the previous work.

The development of absolute pyrometers and the wide application of absolute radiometry to measure radiance temperatures will also affect future agreements on temperature scales. The new, absolute radiometric techniques have been demonstrated to be at least equivalent to the ITS-90 techniques [151, 160], and have the potential to be more accurate, especially at the higher temperatures required in spectroradiometry. The future international temperature scale could include recommendations for absolute radiometric determinations of temperature. Each NMI could maintain a set of standard, absolute pyrometers and the fixed-point black-body sources would serve as check standards. For this to occur, however, it must be established that such a set of standard pyrometers is practical for the intended mode of operation. Based on the results with the irradiance filter radiometers, the absolute radiometric method should result in lower uncertainties for calibration of the typical spectral irradiance and radiance standards compared to the method that utilizes ITS-90 for the temperature determination of the variable-temperature, high-temperature blackbody. With much work, the reduction in the radiance temperature uncertainties at lower temperatures should follow.

## Figures and Tables

**Table 1:** Assigned values of temperatures of fixed points on various International Temperature Scales. The values of temperatures of NHS and ITS-27 are in degrees Centigrade; those of ITS-48 and IPTS-48 are in degrees Celsius; and those of IPTS-68, IPTS-68(75), EPT-76, and ITS-90 are in kelvins

Point	NHS^a^ *t*/°C	ITS-27^b^ *t*/°C	ITS-48^b^ *t*/°C	IPTS-48^b^ *t*/°C	IPTS-68 *T*/K	IPTS-68(75) *T*/K	EPT-76 *T*/K	ITS-90 *T*/K
Cu FP^c^								1357.77
Au FP		1063	1063.0	1063	1337.58	1337.58		1337.33
Ag FP		960.5	960.8	960.8	1235.08	1235.08		1234.93
Al FP								933.473
S BP^d^		444.60	444.600	444.6				
Zn FP				419.505^n^	692.73	692.73		692.677
Sn FP					505.1181^p^	505.1181^p^		505.078
In FP								429.7485
H_2_O BP^e^	100	100.000	100	100	373.15	373.15		
Ga MP^f^								302.9146
H_2_O TP^g^				0.01	273.16	273.16		273.16
H_2_O MP^h^	0	0.000	0					
Hg TP								234.3156
O_2_ BP^i^		−182.97	−182.970	−182.97	90.188	90.188		
Ar TP						83.798^q^		83.8058
O_2_ TP					54.361	54.361		54.3584
Ne BP^j^					27.102	27.102	27.102	
Ne TP							24.5591	24.5561
e-H_2_ BP^k^					20.28	20.28	20.2734	20.3
e-H_2_ BP^l^					17.042	17.042	17.0373	17.0
e-H_2_ TP					13.81	13.81	13.8044	13.8033
Pb SP^m^							7.1999	
^4^He BP							4.2221	4.2
In SP							3.4145	
^3^He BP								3.2
Al SP							1.1796	
Zn SP							0.851	
Cd SP							0.519	

a NHS: Normal hydrogen scale.

b For these temperatures, the ice point was 273.16 °K.

c FP: Freezing point.

d BP: Boiling point at 101 325 Pa.

e H_2_O BP: Steam point.

F MP: Melting point at 101 325 Pa.

g TP: Triple point.

h H_2_O MP: Ice point, saturated with air at 101 325 Pa.

i Redefined in 1975 to condensation point (CP).

j Ne BP: Natural isotopic composition.

k e-H_2_: Equilibrium composition of the ortho/para species.

l Boiling point at reduced pressure, at *p* = 33 330.6 Pa.

m SP: Superconductive transition point.

n Alternative to S BP.

p Alternative to H_2_O BP.

q Alternative to the O_2_ BP.

**Table 2:** NIST fixed-point devices, operating conditions, and measurement uncertainties. The expanded uncertainty (*k* = 2) is denoted by *U*

Fixed point	(mass fraction) %	Container material	Amount of sample	Immersion depth (cm)	Holder material	Furnace or bath	Type A (mK)	Type B (mK)	*U* (mK)
Ag FP	99.9999+	graphite	1.5 kg	13.3	Inconel^a^,^1^	sodium heat pipe	0.50	0.17	1.06
Al FP	99.9999+	graphite	0.4 kg	16.7	Inconel^a^	sodium heat pipe	0.28	0.16	0.64
Zn FP	99.9999+	graphite	1.0 kg	18	glass^b^	three zone	0.18	0.10	0.41
Sn FP	99.9999+	graphite	1.0 kg	18	glass^b^	three zone	0.12	0.02	0.24
In FP	99.9999+	Teflon	1.5 kg	19	ss^d^	three zone	0.04	0.03	0.10
Ga TP	99.99999	Teflon	0.9 kg	13	glass^b^	single zone	0.02	0.01	0.04
H_2_O TP	99.99999	glass^b^	0.50 kg	31.5		maintenance bath	0.003	0.01	0.02
Hg TP	99.999999	glass^b,c^	2.3 kg	17	ss^d^	alcohol bath	0.07	0.01	0.14
Ar TP	99.9999	copper	15 mol	10.9		Dewar	0.03	0.03	0.08

a For protection, the graphite container of Ag and Al are placed inside silica-glass cells before placing in the Inconel holder.

b Borosilicate glass.

c Stainless steel is also used.

d ss: stainless steel.

**Table 3:** Capsule standard platinum resistance thermometer ITS-90 calibrations. Vapor pressure is denoted by VP and the expanded uncertainty (*k* = 2) is denoted by *U*

ITS-90 Fixed Points	e-H_2_ TP	e-H_2_ VP	e-H_2_ VP	Ne TP	O_2_ TP	Ar TP	Hg TP	H_2_O TP	Ga MP	In FP	Sn FP	
ITS-90 assigned temperature (K)	13.8033	17.0	20.3	24.5561	54.3584	83.8058	234.3156	0.01	302.9146	429.7485	505.078	
ITS-90 subranges	*U* (mK)	*U* (mK)	*U* (mK)	*U* (mK)	*U* (mK)	*U* (mK)	*U* (mK)	*U* (mK)	*U* (mK)	*U* (mK)	*U* (mK)	Max *U* (mK)
13.8033 K to 273.16 K	0.22	0.21	0.22	0.26	0.18	0.08	0.20	0.02				0.62
24.5561 K to 273.16 K	0.22			0.26	0.18	0.08	0.20	0.02				0.39
54.3584 K to 273.16 K					0.18	0.08	0.20	0.02				0.29
83.8058 K to 273.16 K						0.08	0.20	0.02				0.39
234.3156 K to 302.9146 K							0.20	0.02	0.04			0.20
273.15 K to 302.9146 K								0.02	0.04			0.04
273.15 K to 429.7485 K								0.02		0.10		0.10
273.15 K to 505.078 K								0.02		0.10	0.24	0.24

**Table 4:** Cryogenic capsule resistance thermometer calibrations. The expanded uncertainty (*k* = 2), is denoted by *U*

Thermometer type	Temperature range (K)	*U* (mK)
RIRTs	0.65 to 24.6	0.46
RIRTs	0.65 to 84	0.46
GRTs	0.65 to 24.6	0.46
GRTs	0.65 to 84	0.46

**Table 5:** Long-stem standard platinum resistance thermometer ITS-90 calibrations. The expanded uncertainty (*k* = 2) is denoted by *U*

ITS-90 Fixed Points	Ar TP	Hg TP	H_2_O TP	Ga MP	In FP	Sn FP	Zn FP	Al FP	Ag FP	
ITS-90 assigned temperature (°C)	−189.3442	−38.8344	0.01	29.7646	156.5985	231.928	419.527	660.323	961.78	
ITS-90 subranges	*U* (mK)	*U* (mK)	*U* (mK)	*U* (mK)	*U* (mK)	*U* (mK)	*U* (mK)	*U* (mK)	*U* (mK)	Max *U* (mK)
−189.3442 °C to 0.01 °C	0.08	0.14	0.02							0.27
−38.8344 °C to 29.7646 °C		0.14	0.02	0.04						0.14
0 °C to 29.7646 °C			0.02	0.04						0.04
0 °C to 156.5985 °C			0.02		0.10					0.10
0 °C to 231.928 °C			0.02		0.10	0.24				0.24
0 °C to 419.527 °C			0.02			0.24	0.41			0.41
0 °C to 660.323 °C			0.02			0.24	0.41	0.64		0.64
0 °C to 961.78 °C			0.02			0.24	0.41	0.64	1.06	1.06

**Table 6:** Industrial platinum resistance thermometer calibrations. The expanded uncertainty (*k* = 2) is denoted by *U*

Comparison calibration							
Comparison bath	LN_2_	Cryostat	Cryostat	Cryostat	Water	Oil	Salt
Temperature range	−196 °C	0 °C to −70 °C	−70 °C to −80 °C	−80 °C to −97 °C	0.5 °C to 95 °C	95 °C to 300 °C	300 °C to 550 °C
*U*(m°C)	2.3	2.3	4	9	2.4	4.8	7.5

Fixed-point calibration			
Fixed point Temperature	ice point 0 °C	H_2_O TP 0.01 °C	Ga MP 29.7646 °C
*U*(m°C)	1.8	1.4	1.6

**Table 7:** Thermocouple thermometer calibrations. The expanded uncertainty (*k* = 2) is denoted by *U*

Thermocouple type	Temperature range (°C)	Type of calibration	*U* (°C)	Thermocouple type	Temperature range (°C)	Type of calibration	*U* (°C)
S	0 to 1100	Fixed point	0.1	E	0 to 1000	Comparison	0.9
S	0 to 1100	Comparison	0.3				
S	1100 to 1450	Extrapolation	1.6	J	0 to 760	Comparison	0.7
R	0 to 1100	Fixed point	0.1	K	0 to 1100	Comparison	1
R	0 to 1100	Comparison	0.3				
R	1100 to 1450	Extrapolation	1.6	N	0 to 1100	Comparison	1
B	0 to 800	Comparison	0.3	T	0 to 400	Comparison	0.4
B	800 to 1100	Comparison	0.3				
B	800 to 1550	Comparison	1.6	All	−196	Comparison	0.4
B	1550 to 1750	Extrapolation	2.4	All	−110 to 315	Comparison	0.4
				All	315 to 550	Comparison	0.5

**Table 8:** Liquid-in-glass thermometer calibrations. The expanded uncertainty (*k* = 2) is denoted by *U*

Thermometer type	Thermomoter liquid	Temperature range (°C)	Thermometer graduation (°C)	*U* (°C)
Total immersion	mercury	0 to 100	0.1	0.02
Total immersion	mercury	0 to 100	0.2	0.02
Total immersion	mercury	100 to 200	0.2	0.06
Total immersion	mercury	200 to 300	0.5	0.05
Total immersion	mercury	300 to 500	1.0	0.16
Total immersion	mercury	−35 to 550	0.1	0.02
Partial immersion	mercury	−35 to 150	0.1	0.1
Partial immersion	mercury	150 to 550	0.1	0.2
Total immersion	organic	−196 to 0	0.1	0.2
Partial immersion	organic	−100 to 0	0.1	0.3

**Table 9:** SRM fixed-point metals. The expanded uncertainty (*k* = 2) is denoted by *U*

SRM number	Metal	Fixed-point temperature (°C)	Unit size (g)	(mass fraction) %	*U* (mK)
743	Hg	−38.8344	680	99.999 999	0.15
1751	Ga	29.7646	200	99.999 995	0.04
1745	In	156.5985	200	99.999 99	0.12
741	Sn	231.928	1300	99.999 9	1.0
741a	Sn	231.928	200	99.999 97	0.25
740	Zn	419.527	350	99.999 9	1.0
740a	Zn	419.527	200	99.999 947	0.7
1744	Al	660.323	200	99.999 96	0.7
1746	Ag	961.78	300	99.999 974	1.1

**Table 10:** Large SRM fixed-point cells. The expanded uncertainty (*k* = 2) is denoted by *U*

SRM 174 fixed-point cell, s/n	Freezing-point (°C)	*U* (mK)	SRM 1748 fixed-point cell, s/n	Freezing-point (°C)	*U* (mK)
Sn 95-1	231.928	0.36	Zn 95-1	419.527	1.01
Sn 95-2	231.928	0.39	Zn 95-2	419.527	1.12
Sn 95-3	231.928	0.37	Zn 95-3	419.527	0.98
Sn 95-4	231.928	0.40	Zn 95-4	419.527	0.94
Sn 95-5	231.928	0.40	Zn 95-5	419.527	1.14

**Table 11:** Small SRM fixed-point cells. The expanded uncertainty (*k* = 2) is denoted by *U*

SRM number	Sample material	Cell type^a^	Fixed-point (C)	Re-entrant well i.d. (mm)	*U* (mK)	Reference number
1968	gallium	MP	29.7646	3.6	0.7	[28]
1972	ethylene carbonate	TP	36.3143	4.5	1.5	[108]
1969	rubidium	TP	39.265	5.0	10	[109,110]
1973	n-docosane	TP	43.879	4.5	2.5	[111]
1970	succinonitrile	TP	58.0642	4.5	1.5	[112–114]
1971	indium	FP	156.5985	4.4	2	[115]

a MP: melting point; FP: freezing point; and TP: triple point.

**Table 12:** SRM thermometers. The expanded uncertainty (*k* = 2) is denoted by *U*

SRM number	Sample material	Temperature range (°C)	Max *U*	Reference number
934	Hg-in-glass thermometer for clinical laboratory	−0.20 to 0.20 and 24 to 34	0.03 K	[116]
1967	Pt thermoelement (Pt-67)	−197 to1768	2μV	[117]
1749	Au/Pt thermocouple	0 to 1000	14 mK	[118]
1750	Capsule SPRT	−259.3467 to 156.5985	0.7 mK	[119]

**Table 13:** A brief summary of radiometric quantities as they apply to non-contact thermometry

Quantity	Quantity symbol	Typical description	SI unit
Power, radiant flux	*ϕ*	Collected by an optical radiation detector	W
Irradiance	*E*	Radiant flux per area at the detector	W m^−2^
Radiance	*L*	Radiant flux in a defined beam and a given direction per area at the source	W m^−2^ sr^−1^
Exitance	*M*	Radiant flux per source area emitted by a source into the hemisphere	W m^−2^
Radiance temperature	*T*_R_	Temperature derived from radiant flux with an emissivity of unity for the source	K

**Table 14:** Values for the constants encountered in radiometry, the standard uncertainties, and the relationship to basic fundamental constants (here *k* is the Boltzmann constant, *ħ* = *h*/(2π), where *h* is the Planck constant, and *c* is the speed of light in vacuum; *c*_1_ = 2π*hc*^2^ is the first radiation constant)

Quantity	Symbol	Expression	Value	SI unit	Rel. Stand. Uncert.
Stefan-Boltzmann constant	*σ*	$\frac{\pi^{2}}{60}\frac{k^{4}}{\hbar^{3}c^{2}}$	5.670 400 × 10^−8^	W m^−2^ K^−4^	7.0 × 10^−6^
First radiation constant for radiance	*c*_1L_	$\frac{c_{1}}{\pi }=2hc^{2}$	1.191 042 722 × 10^−16^	W m^2^ sr^−1^	7.8 × 10^−8^
Second radiation constant	*c*_2_	$\frac{hc}{k}$	0.014 387 752	m·K	1.7 × 10^−6^

**Table 15:** The expanded uncertainties (*k* = 2), in kelvin, for radiance temperature determinations of the blackbodies in the LBIR facility. As the aperture in front of the blackbody becomes smaller, the relative uncertainties become larger due to greater uncertainties in the diffraction correction and geometric alignment uncertainties

Temperature, *T* (K)	Aperture diameter, 2*r*_1_ (mm)				
	0.204	0.284	0.405	3.214	6.407
60				0.16	0.11
70				0.16	0.12
80				0.16	0.12
100				0.17	0.13
125				0.17	0.15
145				0.18	0.16
170			1.4	0.19	0.18
195		2.11	1.38	0.21	0.2
225	2.53	2.1	1.36	0.22	0.22
250	2.56	2.1	1.35	0.24	0.25
275	2.6	2.12	1.35	0.26	0.27
315	2.69	2.16	1.36	0.29	0.3
400	2.95	2.31	1.43	0.35	0.38

**Table 16:** The types of variable-temperature blackbodies available in the LLT facility

Blackbody	Temperature range (°C)	Type of thermometer
Water bath	15 to 90	PRT
Oil bath	90 to 200	PRT
Cs heatpipe	350 to 700	Au/Pt TC
Na heatpipe	600 to 950	Au/Pt TC

**Table 17:** Expanded uncertainty (*k* = 2) in radiance temperature for the Cs or Na pressure-controlled heatpipe blackbody source at 800 °C. The uncertainty is given for two different target diameters of the radiation thermometer

Factor	Radiation thermometer target diameter (mm)	
	1.0	10
BB Emissivity	0.02	0.02
Au/Pt thermocouple	0.01	0.01
Digital voltmeter & ice bath	0.00	0.00
Pressure stability	0.02	0.02
RT noise	0.02	0.02
BB radiance uniformity	0.06	0.57
Total	0.07	0.57

**Table 18:** Expanded uncertainty (*k* = 2) in radiance temperature for an argon-filled ribbon filament lamp in the RTCL. The uncertainty, in °C, is given for different radiance temperatures (also in °C)

Source of Uncertainty	Type	Radiance temperature (°C)				
		800	1100	1500	1900	2300
Calibration of the reference radiance temperature lamp relative to the 1990 NIST Radiance Temperature Scale	B	0.12	0.19	0.32	0.48	0.67
Test lamp temperature determination	A	0.42	0.17	0.29	0.43	0.60
Lamp current measurement	B	0.29	0.19	0.15	0.14	0.14
Mean effective wavelength measurement for the NIST PEP	B	0.10	0.04	0.09	0.28	0.54
Test lamp alignment	B	0.09	0.14	0.24	0.36	0.51
1990 NIST Radiance Temperature Scale relative to thermodynamic temperature scale	B	0.15	0.24	0.40	0.61	0.85
Overall uncertainty of test lamp calibration with respect to SI units	B	0.6	0.4	0.7	1.0	1.5

**Table 19:** Expanded uncertainty (*k* = 2) in radiance temperature for a typical radiation thermometer. The uncertainty, in °C, is given for different radiance temperatures (also in °C)

Source of Uncertainty	Type	Radiance temperature (°C)				
		800	1100	1500	1900	2300
Calibration of the variable temperature blackbody relative to the 1990 NIST Radiance Temperature Scale	B	0.2	0.3	0.4	0.6	0.8
Mean effective wavelength measurement for the NIST PEP	B	0.1	0.0	0.1	0.3	0.5
Blackbody uniformity	B	0.2	0.2	0.2	0.2	0.2
Test thermometer temperature determination	A	0.6	0.6	0.6	0.6	0.6
1990 NIST Radiance Temperature Scale relative to thermodynamic temperature scale	B	0.1	0.2	0.4	0.6	0.9
Overall uncertainty of test radiometer calibration with respect to SI units	B	0.7	0.8	0.9	1.1	1.5

**Table 20:** The component of uncertainty in radiance temperature due to the uncertainty in spectral radiance as a function of wavelength and temperature. A relative expanded uncertainty (*k* = 2) of 0.05 % in spectral radiance was taken for the entire range of parameters. The results are stated in mK (*k* = 2); for parameters that would result in low levels of spectral radiance, no results are given because the measurement precision would be unacceptable

Temperature (K)	Wavelength				
	400 nm	650 nm	900 nm	1500 nm	2000 nm
500				13.0	17.4
1000		22.6	31.3	52.1	69.5
1500	31.3	50.8	70.4	117	156
2000	55.6	90.4	125	209	278
2500	86.9	141	195	326	434
3000	125	203	281	469	626

## Footnotes

1 Certain commercial equipment, instruments, or materials are identified in this paper to foster understanding. Such identification does not imply recommendation or endorsement by the National Institute of Standards and Technology, nor does it imply that the materials or equipment identified are necessarily the best available for the purpose.

2 FEL is a designation, not an acronym.
